# Cortical Spreading Depression Causes Unique Dysregulation of Inflammatory Pathways in a Transgenic Mouse Model of Migraine

**DOI:** 10.1007/s12035-015-9681-5

**Published:** 2016-03-31

**Authors:** Else Eising, Reinald Shyti, Peter A. C. ’t Hoen, Lisanne S. Vijfhuizen, Sjoerd M. H. Huisman, Ludo A. M. Broos, Ahmed Mahfouz, Marcel J. T. Reinders, Michel D. Ferrari, Else A. Tolner, Boukje de Vries, Arn M. J. M. van den Maagdenberg

**Affiliations:** 1 Department of Human Genetics, Leiden University Medical Center, Leiden, The Netherlands; 2 Department of Neurology, Leiden University Medical Center, Leiden, The Netherlands; 3 Department of Intelligent Systems, Faculty of EEMCS, Delft University of Technology, Delft, Netherlands; 4 Division of Image Processing, Department of Radiology, Leiden University Medical Center, Leiden, Netherlands

**Keywords:** Migraine, Familial hemiplegic migraine type 1, Gene expression profiling, Deep serial analysis of gene expression, Inflammation, Interferon

## Abstract

Familial hemiplegic migraine type 1 (FHM1) is a rare monogenic subtype of migraine with aura caused by mutations in *CACNA1A* that encodes the α_1A_ subunit of voltage-gated Ca_V_2.1 calcium channels. Transgenic knock-in mice that carry the human FHM1 R192Q missense mutation (‘FHM1 R192Q mice’) exhibit an increased susceptibility to cortical spreading depression (CSD), the mechanism underlying migraine aura. Here, we analysed gene expression profiles from isolated cortical tissue of FHM1 R192Q mice 24 h after experimentally induced CSD in order to identify molecular pathways affected by CSD. Gene expression profiles were generated using deep serial analysis of gene expression sequencing. Our data reveal a signature of inflammatory signalling upon CSD in the cortex of both mutant and wild-type mice. However, only in the brains of FHM1 R192Q mice specific genes are up-regulated in response to CSD that are implicated in interferon-related inflammatory signalling. Our findings show that CSD modulates inflammatory processes in both wild-type and mutant brains, but that an additional unique inflammatory signature becomes expressed after CSD in a relevant mouse model of migraine.

**Electronic supplementary material:**

The online version of this article (doi:10.1007/s12035-015-9681-5) contains supplementary material, which is available to authorized users.

## Introduction

Migraine is a common brain disorder that is characterised by attacks of severe unilateral headaches accompanied by nausea, vomiting, phono- and/or photophobia [1]. In up to a third of patients, attacks can be accompanied by transient focal neurological aura symptoms that are mostly visual in nature. The likely cause of the aura is cortical spreading depression (CSD), a slow self-propagating wave of neuronal and glial depolarization that originates in the caudal (occipital) cortex and travels frontal through the cortex and is followed by long-lasting temporary neuronal silencing [2, 3]. Experiments in animals revealed that CSD can activate meningeal trigeminovascular neurons and thereby trigeminal ganglia and centres in the brainstem, which confer nociceptive signals further to thalamic and cortical areas that ultimately lead to the sensation of pain [4–8]. Within minutes after CSD, opening of neuronal Pannexin-1 channels triggers an inflammatory cascade in neurons and glial cells that is thought to be responsible for the activation of peripheral and central trigeminovascular neurons [8]. In wild-type mice, CSD causes a pronounced sustained up-regulation of genes involved in inflammation, oxidative stress and intracellular signalling [9–12], but at present, it is unclear to what extent this is also relevant to migraine pathophysiology.

Therefore, we set out to identify changes in gene expression in response to CSD in a transgenic mouse model of familial hemiplegic migraine type 1 (FHM1), which is considered also a relevant model for the common forms of migraine [13]. FHM1 is a rare monogenic subtype of migraine with aura characterised by motor weakness during the aura [1]. FHM1 is caused by mutations in *CACNA1A* gene that codes for the pore-forming α_1A_ subunit of neuronal voltage-gated Ca_V_2.1 channels [14]. Mice that express mutated Ca_V_2.1 channels containing the FHM1 R192Q missense mutation (‘FHM1 R192Q mice’) exhibit an increased susceptibility to CSD due to increased neuronal calcium influx through Ca_V_2.1 channels and increased cortical glutamatergic neurotransmission [15–17]. We used deep serial analysis of gene expression (DeepSAGE) sequencing to identify gene expression changes in the cortex of wild-type (WT) and FHM1 R192Q mice that were subjected to multiple CSD events. To ensure that the gene expression results would not reflect the anaesthesia or surgical procedure, Sham surgery was applied in separate groups of WT and R192Q animals. Tissue was harvested 24 h after the procedure—a time point at which gene expression is known to be affected by CSD [12]—to minimise the effects of the anaesthesia and surgery on gene expression profiles. Several pathway analysis methods were applied to gain insight into the mechanisms that are differentially affected by CSD in the FHM1 R192Q mice compared with WT mice, in order to further our understanding on the role of CSD in migraine pathophysiology.

## Materials and Methods

### Animals

For this study, we used 2- to 4-month-old male WT mice and homozygous transgenic FHM1 mice that carry the human *CACNA1A* R192Q missense mutation (FHM1 R192Q mice). Transgenic mutant mice were generated by introducing the human pathogenic R192Q mutation in the endogenous *Cacna1a* gene using a gene targeting approach [15]. FHM1 R192Q mice of this study were backcrossed to C57BL/6J for six generations. All experimental groups consisted of six mice. Mice were maintained on a normal 12:12 light/dark cycle and water and food were available ad libitum. All procedures were approved by the local Leiden University Medical Center Animal Experiments Ethics Committee.

### Induction of CSD and Tissue Isolation

**Fig. 1:**
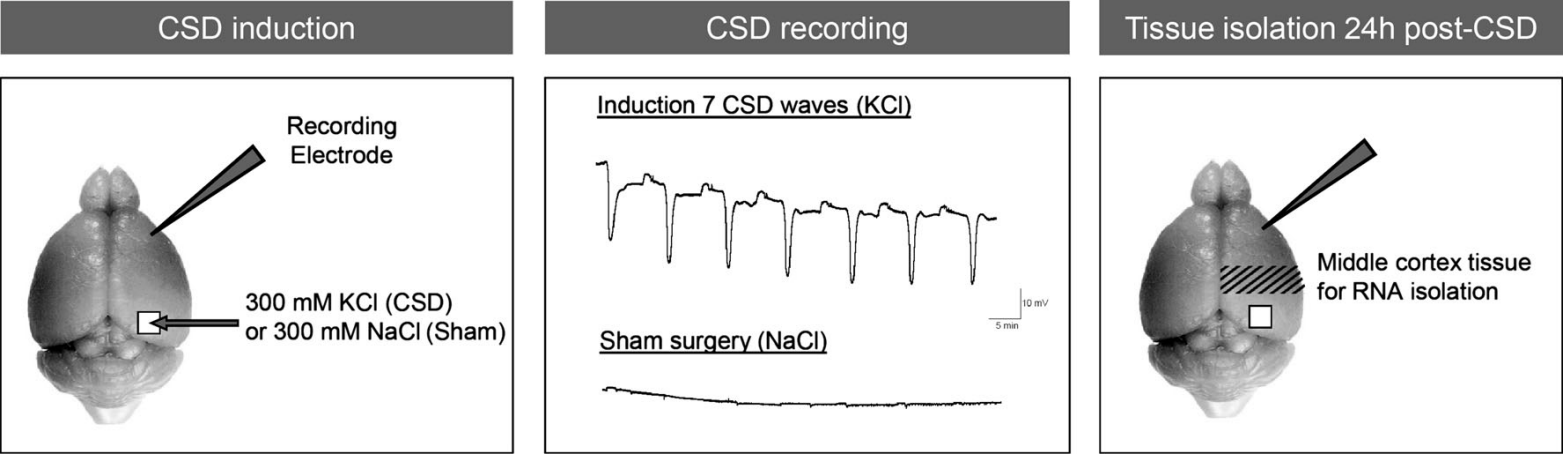
Overview of the experimental procedures for CSD induction, recording and cortical tissue isolation. CSD induction: CSD events were induced under isoflurane anaesthesia by 30-s application of 300 mM KCl (‘CSD-treated’; or 300 mM NaCl for ‘Sham-treated’ groups) on a craniotomy window overlaying the occipital cortex while DC-potential recordings were made from a glass electrode in the frontal cortex. CSD recording: induction of seven CSD waves was obtained by seven applications of KCl, each eliciting a single CSD event in both WT and FHM1 R192Q mutant mice, at 5-min intervals. Sham surgery with application of 300 mM NaCl did not elicit CSD events. Tissue isolation 24 h post-CSD: 24 h after the end of the CSD or Sham procedure brain tissue was isolated. The middle part of the right-sided cortex (including parietal cortex), through which the CSD waves travelled (hashed area), was dissected for RNA profiling

Mice were anaesthetized using 1.5 % isoflurane in pressurised air (20 % O_2_ and 80 % N_2_) and placed onto a stereotactic frame (David Kopf Instruments, Tujunga, CA, USA). Core body temperature was maintained at 37 °C with a heating pad. After exposure of the skull two burr holes were prepared at the following coordinates over the right hemisphere for: (i) CSD induction on the occipital cortex (3.5 mm posterior, 2 mm lateral from bregma) and (ii) CSD recording from the frontal cortex (1.5 mm anterior, 2 mm lateral from bregma). At the recording site in the frontal cortex, a sharp glass capillary electrode (FHC Inc., Bowdoin, ME, USA) filled with 150 mM NaCl was advanced to a depth of 200–300 μm. Data were sampled (200 Hz), amplified (10×) and low-pass filtered at 4 Hz and analysed off-line using LabChart (AD Instruments, Colorado Springs, CO, USA). A reversible DC deflection that was >5 mV in amplitude was considered a CSD event. Seven CSDs were induced by brief application (30 s) of a cotton pellet (Interguide Dental Supply, Burlingame, CA, USA) soaked in 300 mM KCl on the dura overlaying the occipital cortex. Between each KCl application the stimulation area was thoroughly rinsed with saline. The inter-application interval was 5 min; each application induced a single CSD, both in WT and mutant mice. In Sham-treated animals 300 mM NaCl was applied seven times, instead of KCl; NaCl application did not induce CSDs, neither in WT nor in mutant mice. Mice received a subcutaneous injection of 5 mg/kg carprofen for post-operative analgesia after the last CSD or the last NaCl application. Five minutes after the 7th CSD induction or 7th NaCl application, the skin overlaying the skull was sutured and the mice were returned to their home cages to recover from surgery under a heating lamp (37 °C). Twenty-four hours after the end of the CSD or Sham procedure, animals were sacrificed by cervical dislocation. The middle one third of the right hemisphere cortex—through which the CSD waves had passed—that was located between the other thirds that contained either the stimulation (occipital cortex) or the recording (frontal cortex) burr hole was isolated (Fig. 1). After visual inspection to confirm that the cortical tissue did not show obvious damage caused by the surgery or CSD/Sham procedure, tissue was snap frozen in liquid N_2_ within 15 min from the moment of sacrifice, and stored at −80 °C until RNA isolation.

### RNA Isolation

Frozen tissue of the middle third part of the cortex was crunched using a mortar and subsequently homogenised in lysis buffer using the Ultra-turrax T25 Polytron (Janke & Kunkel, Staufen, Germany) mechanical homogeniser. Total RNA was isolated using the Nucleospin RNA II RNA isolation kit (Machery-Nagel, Düren, Germany). Contaminating DNA was removed by on-column treatment with rDNase. Agilent 2100 Bioanalyzer total RNA nanochips (Agilent, Foster City, CA, USA) were used to determine RNA integrity. RNA samples included in this study had RIN (RNA integrity number) values between 8.9 and 10.

### Gene Expression Profiling Using DeepSAGE Sequencing

DeepSAGE libraries were produced and sequenced according to the Illumina protocol by the Leiden Genome Technology Center (LGTC, Leiden, the Netherlands), as described previously [18]. In brief, mRNA was hybridised to Oligo(dT) beads. On the beads, double-stranded complementary DNA (cDNA) was synthesised, which was digested with *Nla*lll and *Mme*l restriction enzymes to create a 17-base-pair (bp) cDNA sequence. The sequence was flanked by two GEX adapters and amplified by PCR for 15 cycles, during which a 6-bp barcode index was introduced that allowed discrimination of reads from up to 12 different samples after sequencing. Library quality and concentration was checked on a high sensitivity DNA assay (Agilent), after which they were sequenced on an Illumina HiSeq2000 sequencer (Illumina, San Diego, CA, USA).

### Processing of the Sequencing Data

The quality of the sequencing results was summarised and plotted using the FASTQ Summary Statistics tool of Galaxy (http://galaxy.psu.edu). Only samples with at least ten million reads were included in the analysis. First, all reads were trimmed to 17 bps to equalise the length of all reads, after which the *Nla*lll recognition site sequence (CATG) was added to the 5′ end of the reads, creating reads with a length of 21 bps. The reads were aligned against the UCSC mm9 mouse genome using Bowtie short read aligner (version 0.12.9), allowing for a maximum of one mismatch and a maximum of one possible position in the genome (options: -k 1 -m 1 -n 1 --best --strata). Mouse exon annotation information was retrieved from BIOMART (Ensembl build 64), and was used to calculate the number of reads per gene for each sample. Only reads aligned to known exons in the sense direction were used for downstream statistical and biological analyses. Analyses were performed at gene level, and reads were summed per gene also when aligned to different locations within a gene, e.g. as a consequence of alternative polyadenylation. Raw gene expression data has been submitted to the Gene Expression Omnibus under accession number GSE67933.

### Statistical Analysis

Statistical analysis of the DeepSAGE sequencing data was performed in R (version 2.15.2) using the Bioconductor package EdgeR (version 3.0.8) [19]. To account for differences in library size, the data was first normalised using the trimmed mean of M-values (TMM) method. A multifactorial generalised linear model was used to calculate the genotype (comparing both FHM1 R192Q and both WT groups) and CSD effects (comparing both CSD-treated with both Sham-treated groups). A second multifactorial model including an intercept term was used to calculate the interaction between genotype and CSD. In both models, the Cox-Reid profile-adjusted likelihood (CR) method was used to estimate the common dispersion. Subsequently, the mean-variance trend was accounted for, and the tag-wise dispersion was calculated as a linear combination of the trended dispersion and the tag-wise dispersion. The amount of shrinkage towards the common dispersion was defined by setting a *prior n* of 10. Finally, differential expression was determined using the generalised linear model likelihood ratio test. For the factors genotype and CSD, as well as for the interaction factor, genes with nominal *p* values ≤0.005 were used for downstream analysis. In an earlier gene expression study in which we compared naïve cortical gene expression levels from FHM1 mice with WT mice, we found an overrepresentation of differentially expressed genes from chromosome 8 resulting from remaining 129/Ola-derived genetic background that was present as a consequence of the gene targeting procedure [20]. Therefore, we removed all genes from chromosome 8 from the genotype effect gene list. Furthermore, genes passing the significance threshold for the interaction factor were removed from the genotype and/or CSD effect lists as they may not reflect a pure genotype or pure CSD effect. Clustering analysis was performed to classify genes according to their expression patterns over the four experimental groups. First, TMM normalised gene expression levels were averaged per experimental group, and the mean and variance were standardised. Next, the expression patterns were clustered by using the k-means method to divide the genes into four groups (settings: number of clusters is ‘4’, method = ‘correlation’).

### Functional Annotation of Gene Sets

To identify overrepresented functional categories in a gene list, gene set enrichment of the PANTHER subset of biological process gene ontology (GO) terms and pathways was performed using DAVID (version 6.7; http://david.abcc.ncifcrf.gov/). Only GO terms and pathways represented by five or more genes from the gene list and with a *p* value <0.05 were considered significant. The STRING database for known and predicted protein-protein interactions (PPIs; version 9.05, http://string-db.org/) [21] was used to identify physical and functional associations between proteins within a gene list. Information from the STRING database based on genomic context, high-throughput experiments, co-expression as well as text mining was used to create protein association networks.

The CORE_TF database for conserved and overrepresented transcription factors (http://grenada.lumc.nl/HumaneGenetica/CORE_TF/) [22] was used to identify transcription factor binding sites in the promoter regions (1000 bp upstream of exon 1) from a gene list. Overrepresentation of transcription factor binding sites was calculated by comparing with a set of 3000 random promoters with similar GC content. Only transcription factors with a binding site frequency of 10 % or more in the random set of promoters were considered. Transcription factor binding sites were linked to transcription factors using information from the molecular signatures database (http://www.broadinstitute.org/gsea/index.jsp). The Interferome database was used to identify interferon-regulated genes in a gene list (version 2.1, standard settings, http://interferome.its.monash.edu.au/interferome/home.jspx) [23].

### Primer Design and Quantitative PCR

An independent set of RNA samples (*N* = 6 for each of the four experimental groups) was used for validation of the DeepSAGE sequencing results by real-time quantitative PCR (RT-qPCR). Upon RNA isolation, first-strand cDNA was synthesised using the RevertAid First-Strand cDNA Synthesis Kit (Thermo Scientific Fermentas, Vilnius, Lithuania). Subsequently, RT-qPCRs were performed on the CFX384 Real-Time PCR Detection System (Bio-Rad, Hercules, CA, USA) using iQ™ SYBR® Green (Bio-Rad) and gene-specific primers (Supplemental Table S1). Primers were designed using Primer 3 software (http://frodo.wi.mit.edu/primer3/) upstream of the DeepSAGE peak or peaks. Genes for the RT-qPCR validation were selected (1) by a high enough expression level for reliable detection by RT-qPCR (which corresponds to >100 raw counts per sample in at least one experimental group) and (2) from the full range of *p* values considered significant (i.e. *p* = 3.4 × 10^−5^ (*Lsg15*) to *p* = 0.0045 (*Ctsz*) for the ‘CSD effect’; 3.48 × 10^−6^ (*Spp1*) to *p* = 0.0031 (*Rsad2*) for cluster 1 of the ‘interaction effect’), which should provide sufficient proof that the results of the whole list are valid. Samples were analysed in duplicate, with *Tbp* and *Gapdh* as housekeeping genes. The RT-qPCR data was analysed with Bio-Rad CFX Manager™ Software (version 3.0). Differential expression was calculated using a one-way analysis of variance (ANOVA) and Bonferroni post-hoc test.

### Comparison with Published Gene Expression Data

Genes with enriched expression in one of the brain cell types (neurons, astrocytes, microglia or oligodendrocytes) under basal conditions were identified using an in-depth literature search [24–31]. To increase the specificity of the gene sets, genes listed more than once were removed. Furthermore, we identified genes differentially expressed after an inflammatory stimulus in brain cell types (neurons, astrocytes and microglia) as well as in macrophages (microglia-related cells). Enrichment of genes from the cell type-enriched or stimulus-related gene lists in cluster 1 (*p* value and odds ratio) was calculated using a Fisher exact test.

### Allen Brain Atlas

For spatial co-expression analysis in the healthy mouse brain we obtained information from RNA in situ hybridisation deposited in the Allen Mouse Brain Atlas (http://www.brain-map.org/) [32]. The data set contains probe expression values summarised at the voxel level. Selection of only data of the left hemisphere and filtering out voxels with more than 75 % missingness left 27,365 voxels and 26,022 probes of 19,909 genes. To obtain a gene co-expression network, cosine similarities were calculated between all probes and the mean similarity per gene pair was taken. Of the 96 genes in cluster 1, 87 genes could be matched to Allen Brain Atlas data. To test whether the spatial co-expression of cluster 1 was significantly different from random gene sets, a random permutation test was performed by drawing gene sets of the same size and constructing a null distribution of the mean co-expression value.

## Results

### DeepSAGE Sequencing in Cortical Mouse Brain Samples that Were Subjected to Cortical Spreading Depression

The cortical transcriptome of FHM1 R192Q mutant and WT mice was investigated using DeepSAGE sequencing, 24 h after experimental induction of CSD or Sham surgery (*N* = 6/experimental group) (Fig. 1). Over 10 million high-quality sequencing reads were obtained per sample (median per base phred score was 31 or higher) of which on average 52 % (range 45–57 %) could be uniquely aligned to known exons in the mouse genome (Supplemental Table S2). Similar numbers of total reads and aligned reads were observed for the four experimental groups (Supplemental Table S2). For each sample, the expression level per gene was calculated. A total of 23,847 mouse genes could be detected in our dataset.

### Cortical Spreading Depression Affects Expression of Immune System-Related Genes

Cortical gene expression levels were compared between FHM1 R192Q mutant and WT mice (‘genotype effect’) and between the CSD- and Sham-treated groups (CSD effect). Gene expression changes were modest. Therefore, we used a less-stringent threshold of uncorrected *p* < 0.005 and focused our downstream analyses and interpretation of the data on gene sets rather than individual genes to diminish the contribution of false positives. Furthermore, extra effort was put in validating promising results with RT-qPCR in an independent group of animals. Only 45 genes showed a genotype effect (Supplemental Table S3). The CSD effect was more pronounced with 80 genes showing unadjusted *p* < 0.005 (Supplemental Table S4). A GO term enrichment analysis of the CSD effect showed that biological functions and pathways affected by CSD in the mice are largely involved in immune system-related functions (Table 1).

**Table 1:** GO terms and pathways significantly overrepresented in the ‘CSD effect’ gene set

Category	Term	*p* value
PANTHER_BP_ALL	BP00156: interferon-mediated immunity	6.12 × 10^−5^
PANTHER_BP_ALL	BP00148: immunity and defence	5.85 × 10^−4^
PANTHER_BP_ALL	BP00107: cytokine and chemokine mediated signalling pathway	2.14 × 10^−3^
PANTHER_MF_ALL	MF00173: defence/immunity protein	0.01

Gene expression differences were validated by RT-qPCR in biologically independent samples (*N* = 6/experimental group) for a selection of genes that showed a genotype effect or a CSD effect (Supplemental Figs. S1 and S2). Since the majority of the 45 genes that showed a genotype effect had a very low expression level, we could select only three for validation with RT-qPCR. Only one of them, *Klk6*, showed a significant difference in expression level based on ANOVA testing, whereas Bonferroni post hoc testing did not show differences between both FHM1 R192Q mutant and WT groups, as would be expected for a true difference between genotypes (Supplemental Fig. S1). From the CSD effect set of 80 genes, eight were selected for validation by RT-qPCR from which six, *Isg151*, *Cd180*, *Trim30a*, *Bst2*, *Samd9l* and *Ctsz*, showed significant differential expression in the RT-qPCR validation (Supplemental Fig. S2).

### Interaction Analysis Identifies Up-regulation of Interferon-Related Genes After Cortical Spreading Depression, Specifically in the Brains of FHM1 R192Q Mutant Mice

**Fig. 2:**
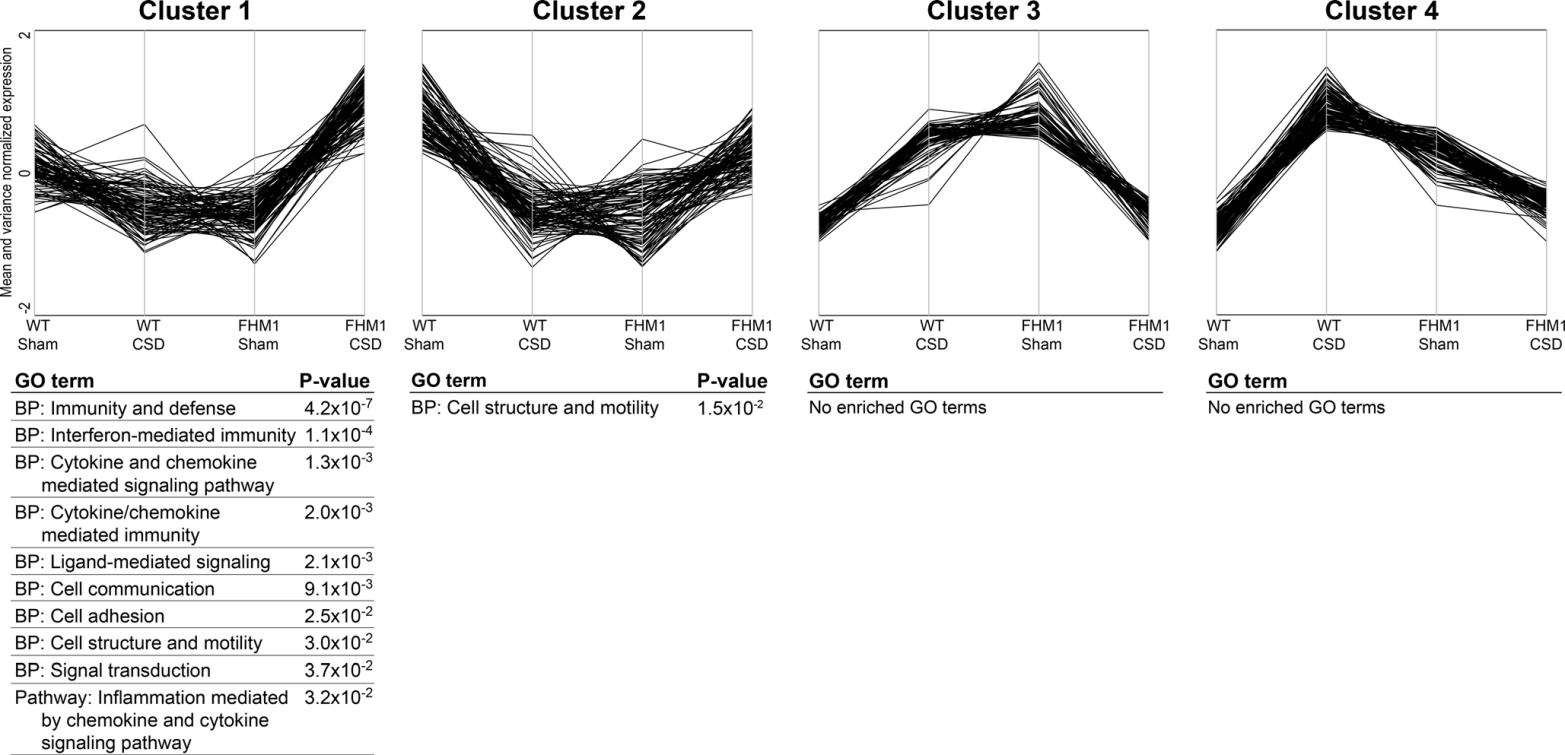
K-means clustering of the genes from the ‘interaction effect’. Mean- and variance-normalised gene expression data of the 360 genes from the interaction effect were clustered into four clusters with different expression patterns over the four experimental groups (WT Sham-treated, WT CSD-treated, FHM1 Sham-treated and FHM1 CSD-treated). Below each cluster, enriched GO terms and their respective *p* values are shown. The GO term enrichment was generated using DAVID, which classifies genes for biological process (*BP*) and pathways from the PANTHER subset of GO terms

A multifactorial model that included an interaction factor was used to identify genes that responded differently to CSD in FHM1 R192Q mutant and WT mice. Some 360 genes demonstrated uncorrected *p* values <0.005 for the interaction factor (interaction effect) (Supplemental Table S5). In order to identify sets of genes with similar expression patterns, genes were classified into four clusters based on their expression levels in the four experimental groups using k-means cluster analysis (Fig. 2; Supplemental Table S5).

**Fig. 3:**
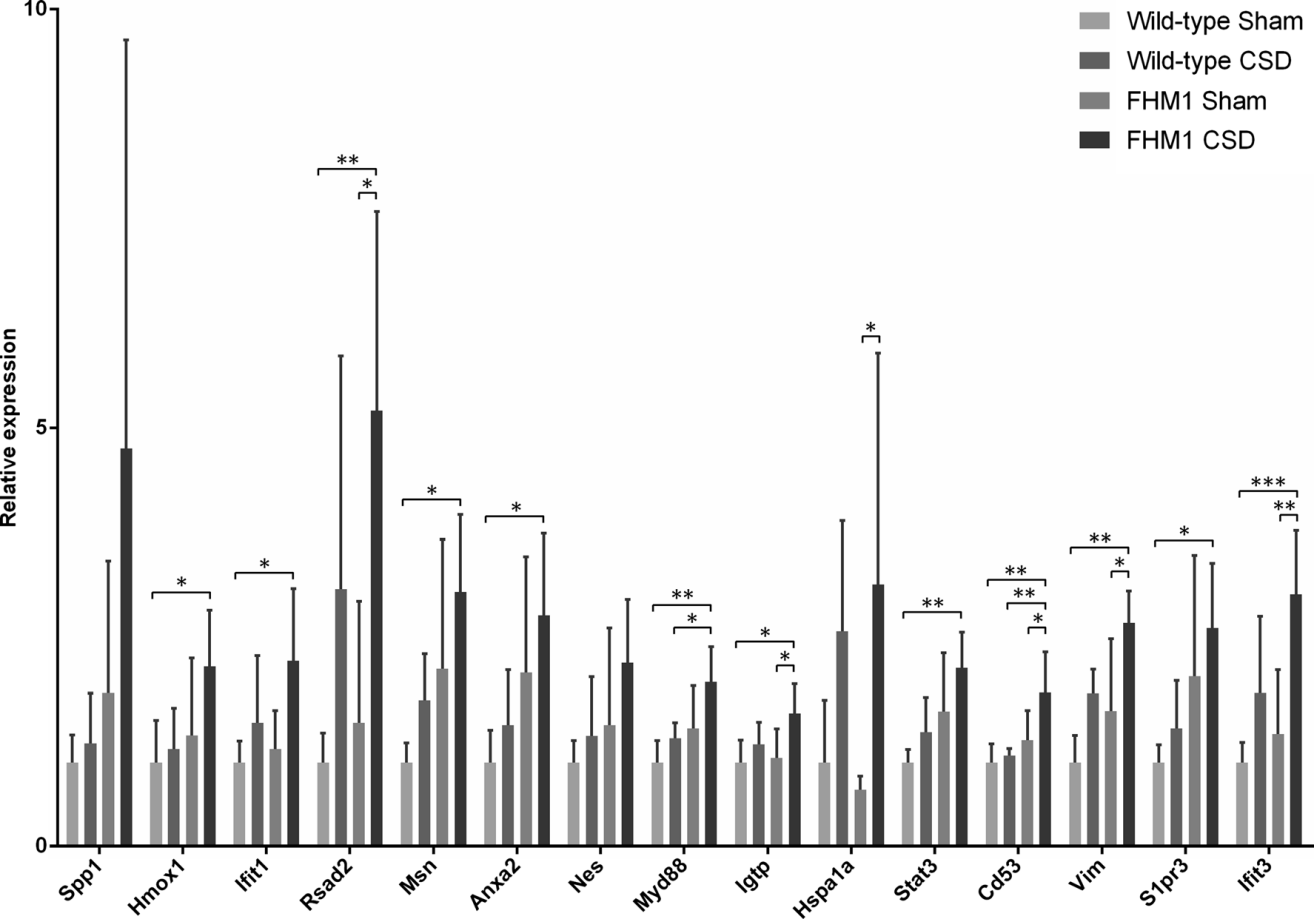
RT-qPCR validation of cluster 1 in biologically independent samples. The selected genes from cluster 1 were all present in the protein interaction network. Data were normalised to *Tbp* and *Gapdh* mRNA expression and expressed as fold-changes relative to the WT Sham-treated group (means ± SD). *p < 0.05; **p < 0.01; ***p < 0.001 according to a one-way ANOVA with Bonferroni post-hoc test

Subsequently, a GO term and pathway analysis was performed on the genes from each cluster to determine whether genes with similar expression patterns also have similar functions (Fig. 2). Only cluster 1 showed a clear enrichment of functional categories. The expression levels of the 95 genes within this cluster were increased after CSD induction; more strongly in brains of FHM1 R192Q mutant mice. GO term analysis indicated that many genes from cluster 1 are involved in immune system responses with several having a function in the regulation of cell adhesion and motility. GO terms included ‘immunity and defence’ (*p* = 2.4 × 10^−7^), ‘interferon-mediated immunity’ (*p* = 1.1 × 10^−4^) and ‘cytokine- and chemokine-mediated signalling pathway’ (*p* = 1.3 × 10^−3^). A selection of genes from cluster 1 was used for the validation of the initial results by RT-qPCR in the set of independent biological samples. Thirteen out of 15 analysed genes showed significant differential expression between the four experimental groups as measured with an ANOVA test (Fig. 3). Most striking, all genes showed highest expression in the FHM1 R192Q mutant CSD group, thereby replicating and validating the DeepSAGE sequencing data.

**Fig. 4:**
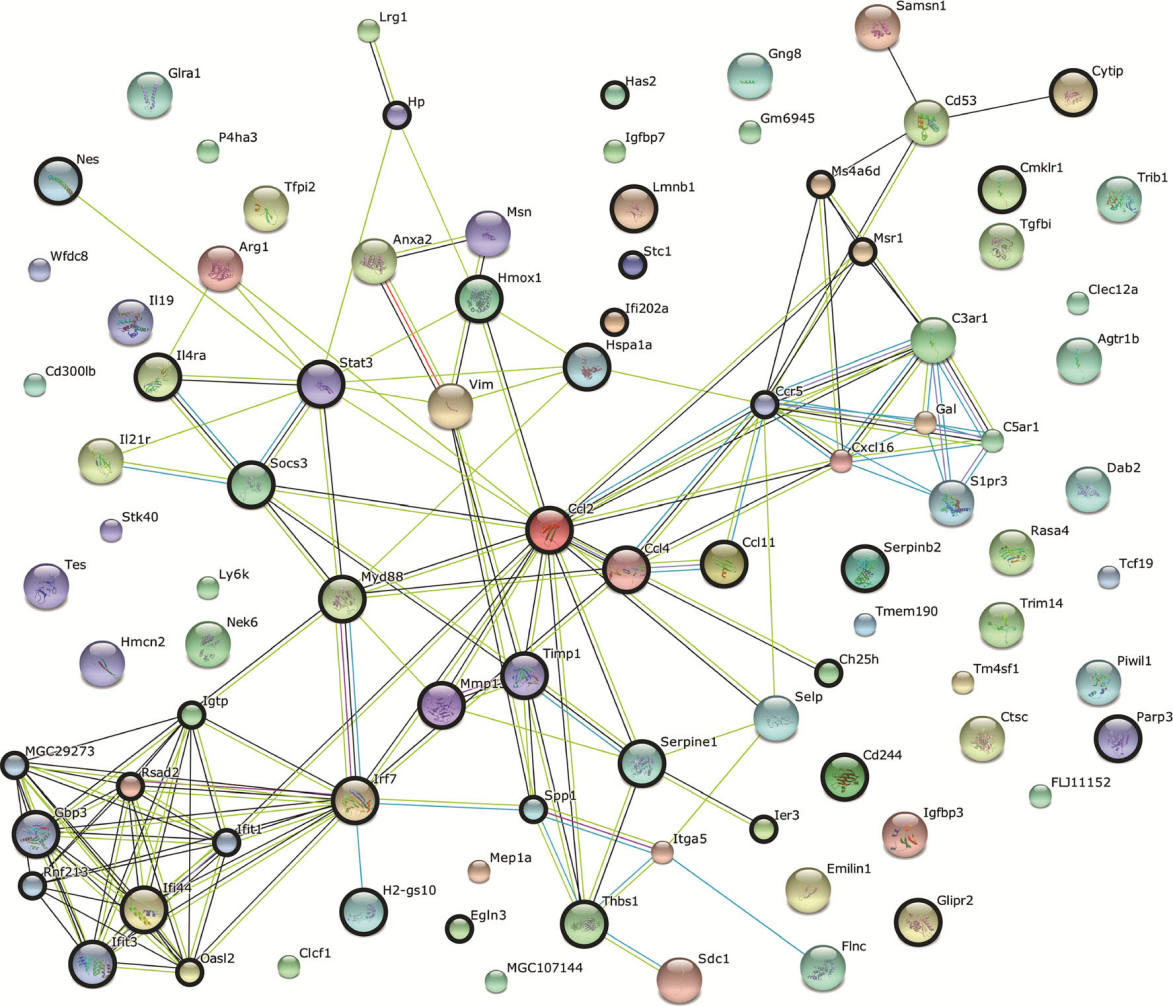
Protein association network in cluster 1. The protein association network is derived from the STRING database. The nodes represent the genes from cluster 1. The connections between the nodes represent the interactions based on co-expression (*black*), text mining (*light green*) association in curated database (*light blue*) and high-throughput experiments (*pink*). *Nodes with bold circles* represent interferon-responsive genes from the Interferome database

To further explore the genes from cluster 1, the STRING database for protein associations was used, which showed that 49 genes from this cluster (52 %) group together in a significant protein association network largely based on co-expression data and text mining (Fig. 4). Text mining associations from the protein association network indicated that many genes from cluster 1 are involved in interferon-mediated signalling. Therefore, we compared the genes from cluster 1 to the Interferome database of interferon-regulated genes and found that a striking 43 of the 95 genes from cluster 1 (44 %) are annotated as interferon-regulated genes (Fig. 4). To further identify possible signalling cascades that could have regulated the expression pattern of genes from cluster 1, a transcription factor binding site overrepresentation analysis was performed that revealed that binding sites of 15 transcription factors are overrepresented in the promoter regions of the genes from cluster 1, including the interferon regulatory factors 1 and 8 and STAT1/2 that are involved in interferon signalling (Table 2).

**Table 2:** Transcription factor binding site overrepresentation in cluster 1

TF binding site	Transcription factor	Study freq.	Random freq.	*p* value
IRF_Q6 ^a^	Interferon regulatory factor 1	0.20	0.12	6.41 × 10^−3^
KAISO_01	Zinc finger and BTB domain containing 33	0.38	0.27	7.79 × 10^−3^
NFE2_01	Nuclear factor, erythroid derived 2	0.34	0.24	7.82 × 10^−3^
BACH2_01	BTB and CNC homology 2	0.16	0.09	8.41 × 10^−3^
YY1_01	YY1 transcription factor	0.98	0.93	9.29 × 10^−3^
CACBINDING-PROTEIN_Q6	Not matched to known transcription factor	0.74	0.64	0.01
ISRE_01 ^a^	Signal transducer and activator of transcriptions 1 and 2	0.19	0.12	0.01
AP1_01	Jun oncogene	0.96	0.90	0.01
ICSBP_Q6 ^a^	Interferon regulatory factor 8	0.16	0.10	0.01
XFD3_01	Forkhead box A2	0.93	0.87	0.01
STAT5B_01	Signal transducer and activator of transcription 5B	0.29	0.20	0.02
TFIII_Q6	General transcription factor IIA, 1 and 2	0.82	0.75	0.04
AP4_01	Transcription factor AP-4	0.38	0.31	0.05
MAZ_Q6	MYC-associated zinc finger protein (purine-binding transcription factor)	0.51	0.43	0.05
HTF_01	X-box binding protein 1	0.50	0.42	0.05

^a^Transcription factors and their binding sites directly involved in interferon-mediated signalling

### Genes from Cluster 1 Are Not Co-expressed in Naïve Mouse Brain

**Fig. 5:**
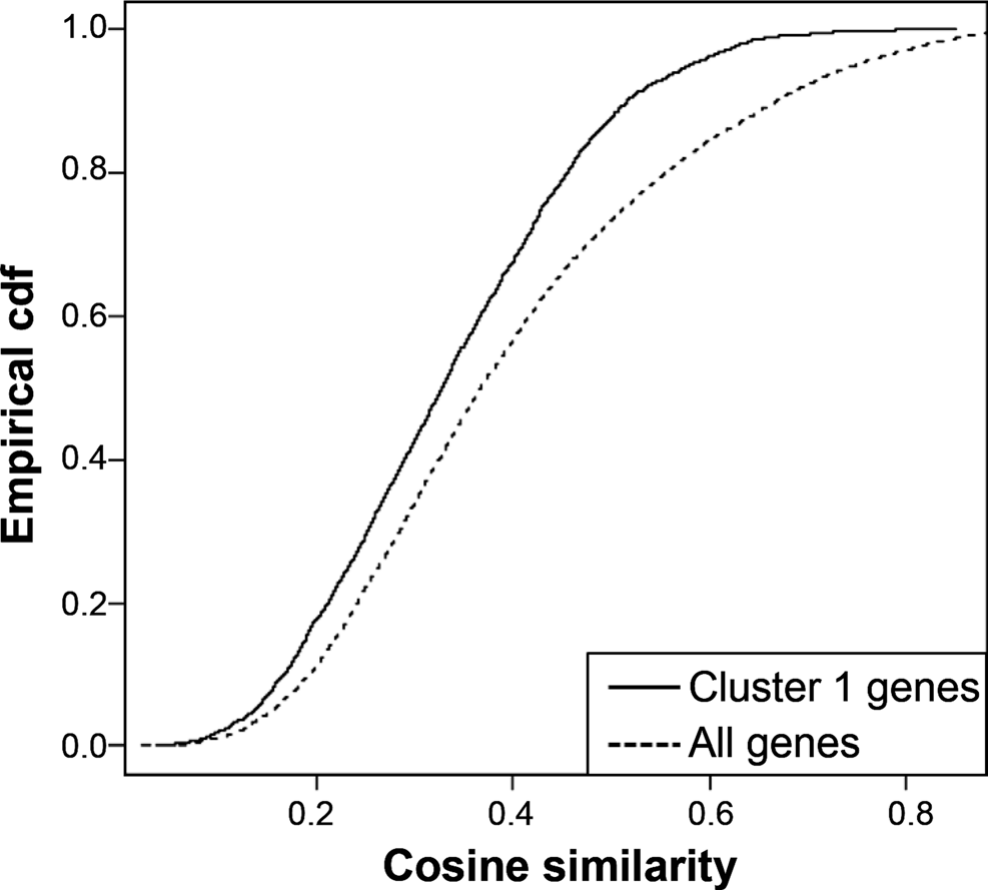
Co-expression analysis of cluster 1 genes in the naïve mouse brain. The naïve mouse brain expression data were retrieved from the Allen Brain Atlas. Genes with a high cosine similarity have similar spatial expression patterns. Cosine similarities between genes from cluster 1 are compared to those between randomly drawn gene sets of the same size, using their cumulative density functions. The mean similarity between cluster 1 genes is significantly lower than that of randomly drawn gene sets. *Cdf* cumulative distribution function

The protein association network analysis (Fig. 4) showed that many genes from cluster 1 are co-expressed, but does not specify whether the co-expression concerns a certain tissue or cell type. Information from the Allen Brain Atlas gene expression database was used to calculate the level of co-expression of genes from cluster 1 in the naïve adult mouse brain. Genes from cluster 1 appear to be significantly less spatially co-expressed than random genes in the healthy mouse brain (*p* = 0.003) (Fig. 5), in line with the lower expression of the genes in the naïve brain. To identify cell types that could be involved in the inflammatory response upon CSD in our mice, genes from cluster 1 were compared with published gene lists containing neuron-, astrocyte-, microglia- or oligodendrocyte-enriched genes (Table 3) [24–26]. A smaller than expected number of neuron- and oligodendrocyte-enriched genes was found in cluster 1. Next, the gene list of cluster 1 was compared with a lists of genes that appeared differentially expressed after treatment with an inflammatory trigger in several brain cell types and macrophages [25, 27–31]. A significantly higher than expected number of 39 genes out of 95 of cluster 1 overlap with this gene list (*p* = 2.20 × 10^−16^) (Table 3).

**Table 3:** Cell type specificity of genes from cluster 1

	Total count	Study count	*p* value	Odds ratio	References
Neuron-enriched genes	2436	3	0.01	0.27	[24, 26]
Astrocyte-enriched genes	2261	13	0.23	1.43	[24, 26]
Microglia-enriched genes	85	2	0.05	5.80	[25, 26]
Oligodendrocyte-enriched genes	1932	2	0.02	0.23	[24, 26]
Immune stimulation-responsive genes	1647	39	2.20 × 10^−16^	8.88	[25, 27–31]

## Discussion

Here we investigated the molecular effects of CSD, as a surrogate for migraine with aura, on gene expression level in a well-validated migraine mouse model [13]. Gene expression changes were investigated 24 h after CSD induction (or Sham treatment)—to avoid confounding influences of the surgery and anaesthesia—in cortical tissue of FHM1 R192Q mutant mice and WT mice to assess whether long-lasting and/or delayed changes in molecular mechanisms occurred in response to CSD. Three sets of differentially expressed genes were identified: (i) genes with differential expression between genotypes (genotype effect); (ii) genes with differential expression between CSD induction and Sham surgery (CSD effect); and (iii) genes that show an interaction between genotype and CSD induction (interaction effect).

The genotype effect was only limited. This is in line with our previous study that identified only minor differences in cortical gene expression between FHM1 mice and WT under naïve conditions [20]. Also, the CSD effect was relatively small, which shows that the brain is relatively resistant with respect to changing the expression of genes even after a major perturbation such as multiple CSD events. The inflammation-related genes in the CSD effect are not unexpected, as CSD induction is known to activate inflammatory signalling cascades already within minutes [8] but also with a time delay of hours that can last for days [9–12]. In addition, CSD induction is known to cause long-term activation of microglia [33]. As for our gene expression analysis, we only investigated the 24 h time point after CSD. It needs to be established whether additional CSD-induced FHM1-specific gene expression changes occur at later time points. This may well be the case as certain inflammatory genes such as *Cd74*, *Lgals3* and *Timp1* [12] have been shown to take longer to become expressed or up-regulated upon experimentally induced spreading depression. Moreover, (presumed) downstream effects of inflammatory changes related to myelin were observed up to 3 days following SD [34] and related to activation of brain regions 40 h following CSD [35]. Still, since a microarray gene expression study investigating WT rats that underwent CSD showed less profound changes at time points later than 24 h following CSD [12], we feel that the 24 h time point sufficiently captured relevant gene expression differences induced by CSD. The observation of an up-regulation of inflammatory genes after CSD in comparison to the Sham-treated group suggests minor influence of surgical procedures or post-surgery analgesia.

The most remarkable finding of the study was the up-regulation of a set of genes after CSD specifically in FHM1 R192Q mutant brains (cluster 1 from the interaction effect). These genes show a strong functional enrichment for genes involved in inflammatory signalling. Several genes in cluster 1 (*Cd53*, *Ms4a6d*, *Anxa2*, *Ccl2*, *Vim*, *C3ar1* and *Timp1*) are key drivers of inflammatory reactions [36]. The high abundance of genes from cluster 1 in the Interferome database of interferon-regulated genes, as well as the overrepresentation of interferon-related transcription factor binding sites (IRF, ISRE and ICSBP) in the promoter regions of these genes, indicate a major role for interferon-mediated inflammatory signalling in the response to CSD that is specific for FHM1 R192Q mutant mice. A clear role for interferon-related responses in migraine pathophysiology has not yet been identified.

Remarkably, genes from cluster 1 showed *reduced* co-expression in the naïve mouse brain but *increased* co-expression in brain-related cell types that were subjected to a strong, inflammatory, stimulus. This suggest that the up-regulation of genes in cluster 1 is truly stimulus dependent. Notably, a meta-analysis of gene expression studies of respiratory virus infections revealed that expression of multiple genes from cluster 1, e.g. *Ifit3*, *Tgfbi* and *Igtp,* is positively correlated with the severity of infection [37], suggesting that these genes are markers of severity of inflammatory responses. Furthermore, 11 out of 95 genes from cluster 1 have been found to be differentially expressed also upon induction in experimental animals of status epilepticus and traumatic brain injury; two widely used models with triggers that should be considered more severe than CSD [38]. The altered expression of cluster 1 genes could therefore reflect that CSD might evoke a more dramatic response in the brains of FHM1 R192Q mutant mice than in WT mice. The dramatic response seems a direct consequence of the presence of mutated Ca_V_2.1 calcium channels [15, 17]. Future studies in FHM1 S218L mice, which express a more severe mutation that results in an even more enhanced gain-of-function of CaV2.1 channels and CSD susceptibility with additional neurological symptoms including seizures and cerebellar ataxia [39], may address whether the identified changes in gene expression in FHM1 R192Q are more pronounced when Ca_V_2.1 channel activity is more affected. Also, due to the associated neurological deficits additional gene pathways may be revealed.

How this interaction between CSD and the FHM1 R192Q mutation would result in a specific inflammatory profile remains to be identified. Studies in epilepsy models already hinted at a close interplay between enhanced excitatory activity and inflammation [40, 41], which suggests that the enhanced glutamatergic activity in FHM1 R192Q mutant mice [17] that is more pronounced during CSD episodes may trigger a particularly severe inflammatory response. Alternatively, one can speculate that the enhanced inflammatory response to CSD may be the result of a pro-inflammatory state in the brains of FHM1 R192Q mutant mice that may already exist in naïve animals. In that respect, it is noteworthy that naïve trigeminal ganglia of FHM1 R192Q migraine mice exhibit a pro-inflammatory phenotype with a higher number of activated macrophages, activated microglia and increased cytokine expression levels [42–45]. In the caudal cortex, in which CSD events originate, only nine genes are differently expressed in naïve FHM1 mutant mice [20]. Of those genes, *Camkk1, Gpr34, Tom1l2* and *Cort* are linked to inflammation, thus providing some evidence that a pro-inflammatory state may also exist in the naïve FHM1 R192Q cortex. Finally, changes in vascular permeability and possible intrusion of white blood cells might be involved in the inflammatory state of the brain after CSD induction. CSD has previously been shown to cause opening of the blood brain barrier (BBB) through activation of the proteinase MMP9 in rats [46]. In addition, in rat hippocampal slices, spreading depression was shown to cause abnormal interferon signalling through activation of T cells [34]. Although the CSD effect and cluster 1 of the interaction effect do not contain markers for white blood cells, increased BBB permeability and possibly subsequent white blood cell intrusion may provide an attractive explanation for the enhanced interferon-related inflammatory signature in FHM1 R192Q mice after CSD. The identification of an increased specific inflammatory response after CSD in a relevant migraine mouse model raises the question whether CSD in FHM1 R192Q mice may lead to an increased activation of meningeal nociceptors and trigeminal ganglia, which would drive the activation of pain-related brain structures ultimately causing migraine headache [6–8].

In summary, CSD caused a specific inflammatory signalling response in the cortex of FHM1 R192Q mutant mice. First evidence now emerges that a possible pro-inflammatory state in the naïve brain of FHM1 R192Q mice may lead to an exacerbated inflammatory reaction, in response to a strong stimulus like CSD. Pathway analyses suggest that this exacerbated inflammatory reaction is interferon related. This study suggests that interferon plays an important role in the effects of CSD and reinforces the concept that neuro-inflammation may play an important role in migraine pathophysiology.

## Electronic Supplementary Material

Below is the link to the electronic supplementary material.

ESM 1 (PDF 454 kb)

ESM 2 (PDF 810 kb)

## References

[1] Headache Classification Committee of the International Headache Society (IHS) (2013). The International Classification of Headache Disorders, 3rd edition (beta version). Cephalalgia.

[2] Lauritzen M (1994). Pathophysiology of the migraine aura. The spreading depression theory. Brain.

[3] Hadjikhani N, Sanchez Del Rio M, Wu O, Schwartz D, Bakker D, Fischl B, Kwong KK, Cutrer FM, Rosen BR, Tootell RB (2001). Mechanisms of migraine aura revealed by functional MRI in human visual cortex. Proc Natl Acad Sci U S A.

[4] Bolay H, Reuter U, Dunn AK, Huang Z, Boas DA, Moskowitz MA (2002). Intrinsic brain activity triggers trigeminal meningeal afferents in a migraine model. Nat Med.

[5] Noseda R, Burstein R (2013). Migraine pathophysiology: anatomy of the trigeminovascular pathway and associated neurological symptoms CSD sensitization and modulation of pain. Pain.

[6] Zhang X, Levy D, Kainz V, Noseda R, Jakubowski M, Burstein R (2011). Activation of central trigeminovascular neurons by cortical spreading depression. Ann Neurol.

[7] Zhang X, Levy D, Noseda R, Kainz V, Jakubowski M, Burstein R (2010). Activation of meningeal nociceptors by cortical spreading depression: implications for migraine with aura. J Neurosci.

[8] Karatas H, Erdener SE, Gursoy-Ozdemir Y, Lule S, Eren-Kocak E, Sen ZD, Dalkara T (2013). Spreading depression triggers headache by activating neuronal Panx1 channels. Science.

[9] Jander S, Schroeter M, Peters O, Witte OW, Stoll G (2001). Cortical spreading depression induces proinflammatory cytokine gene expression in the rat brain. J Cereb Blood Flow Metab.

[10] Choudhuri R, Cui L, Yong C, Bowyer S, Klein RM, Welch KM, Berman NE (2002). Cortical spreading depression and gene regulation: relevance to migraine. Ann Neurol.

[11] Thompson CS, Hakim AM (2005). Cortical spreading depression modifies components of the inflammatory cascade. Mol Neurobiol.

[12] Urbach A, Bruehl C, Witte OW (2006). Microarray-based long-term detection of genes differentially expressed after cortical spreading depression. Eur J Neurosci.

[13] Ferrari MD, Klever RR, Terwindt GM, Ayata C, van den Maagdenberg AMJM (2015). Migraine pathophysiology: lessons from mouse models and human genetics. Lancet Neurol.

[14] Ophoff RA, Terwindt GM, Vergouwe MN, van Eijk R, Oefner PJ, Hoffman SM, Lamerdin JE, Mohrenweiser HW, Bulman DE, Ferrari MD (1996). Familial hemiplegic migraine and episodic ataxia type-2 are caused by mutations in the Ca2+ channel gene CACNL1A4. Cell.

[15] van den Maagdenberg AMJM, Pietrobon D, Pizzorusso T, Kaja S, Broos LA, Cesetti T, van de Ven RC, Tottene A, van der Kaa J, Plomp JJ (2004). A Cacna1a knockin migraine mouse model with increased susceptibility to cortical spreading depression. Neuron.

[16] Eikermann-Haerter K, Dilekoz E, Kudo C, Savitz SI, Waeber C, Baum MJ, Ferrari MD, van den Maagdenberg AMJM, Moskowitz MA, Ayata C (2009). Genetic and hormonal factors modulate spreading depression and transient hemiparesis in mouse models of familial hemiplegic migraine type 1. J Clin Invest.

[17] Tottene A, Conti R, Fabbro A, Vecchia D, Shapovalova M, Santello M, van den Maagdenberg AMJM, Ferrari MD, Pietrobon D (2009). Enhanced excitatory transmission at cortical synapses as the basis for facilitated spreading depression in Ca(v)2.1 knockin migraine mice. Neuron.

[18] t Hoen PA, Ariyurek Y, Thygesen HH, Vreugdenhil E, Vossen RH, de Menezes RX, Boer JM, van Ommen GJ, den Dunnen JT (2008) Deep sequencing-based expression analysis shows major advances in robustness resolution and inter-lab portability over five microarray platforms. Nucleic Acids Res 36:e141 10.1093/nar/gkn705 PMC2588528 18927111

[19] Robinson MD, McCarthy DJ, Smyth GK (2010). edgeR: a Bioconductor package for differential expression analysis of digital gene expression data. Bioinformatics.

[20] de Vries B, Eising E, Broos LA, Koelewijn SC, Todorov B, Frants RR, Boer JM, Ferrari MD, Hoen tPA, van den Maagdenberg AMJM (2014). RNA expression profiling in brains of familial hemiplegic migraine type 1 knock-in mice. Cephalalgia.

[21] Franceschini A, Szklarczyk D, Frankild S, Kuhn M, Simonovic M, Roth A, Lin J, Minguez P, Bork P, von Mering C (2013). STRING v9.1: protein-protein interaction networks with increased coverage and integration. Nucleic Acids Res.

[22] Hestand MS, van Galen M, Villerius MP, van Ommen GJ, den Dunnen JT, Hoen tPA (2008). CORE_TF: a user-friendly interface to identify evolutionary conserved transcription factor binding sites in sets of co-regulated genes. BMC Bioinformatics.

[23] Rusinova I, Forster S, Yu S, Kannan A, Masse M, Cumming H, Chapman R, Hertzog PJ (2013). Interferome v2.0: an updated database of annotated interferon-regulated genes. Nucleic Acids Res.

[24] Cahoy JD, Emery B, Kaushal A, Foo LC, Zamanian JL, Christopherson KS, Xing Y, Lubischer JL, Krieg PA, Krupenko SA (2008). A transcriptome database for astrocytes neurons and oligodendrocytes: a new resource for understanding brain development and function. J Neurosci.

[25] Glanzer JG, Enose Y, Wang T, Kadiu I, Gong N, Rozek W, Liu J, Schlautman JD, Ciborowski PS, Thomas MP (2007). Genomic and proteomic microglial profiling: pathways for neuroprotective inflammatory responses following nerve fragment clearance and activation. J Neurochem.

[26] Kang HJ, Kawasawa YI, Cheng F, Zhu Y, Xu X, Li M, Sousa AM, Pletikos M, Meyer KA, Sedmak G (2011). Spatio-temporal transcriptome of the human brain. Nature.

[27] Hamby ME, Coppola G, Ao Y, Geschwind DH, Khakh BS, Sofroniew MV (2012). Inflammatory mediators alter the astrocyte transcriptome and calcium signaling elicited by multiple G-protein-coupled receptors. J Neurosci.

[28] Lisak RP, Nedelkoska L, Studzinski D, Bealmear B, Xu W, Benjamins JA (2011). Cytokines regulate neuronal gene expression: differential effects of Th1 Th2 and monocyte/macrophage cytokines. J Neuroimmunol.

[29] Paglinawan R, Malipiero U, Schlapbach R, Frei K, Reith W, Fontana A (2003). TGFbeta directs gene expression of activated microglia to an anti-inflammatory phenotype strongly focusing on chemokine genes and cell migratory genes. Glia.

[30] Wang J, Campbell IL (2005). Innate STAT1-dependent genomic response of neurons to the antiviral cytokine alpha interferon. J Virol.

[31] Zhang S, Kim CC, Batra S, McKerrow JH, Loke P (2010). Delineation of diverse macrophage activation programs in response to intracellular parasites and cytokines. PLoS Negl Trop Dis.

[32] Lein ES, Hawrylycz MJ, Ao N, Ayres M, Bensinger A, Bernard A, Boe AF, Boguski MS, Brockway KS, Byrnes EJ (2007). Genome-wide atlas of gene expression in the adult mouse brain. Nature.

[33] Grinberg YY, Milton JG, Kraig RP (2011). Spreading depression sends microglia on Lévy flights. PLoS One.

[34] Pusic AD, Mitchell HM, Kunkler PE, Klauer N, Kraig RP (2015). Spreading depression transiently disrupts myelin via interferon-gamma signaling. Exp Neurol.

[35] Cui Y, Toyoda H, Sako T, Onoe K, Hayashinaka E, Wada Y, Yokoyama C, Onoe H, Kataoka Y, Watanabe Y (2015). A voxel-based analysis of brain activity in high-order trigeminal pathway in the rat induced by cortical spreading depression. Neuroimage.

[36] Wang IM, Zhang B, Yang X, Zhu J, Stepaniants S, Zhang C, Meng Q, Peters M, He Y, Ni C (2012). Systems analysis of eleven rodent disease models reveals an inflammatome signature and key drivers. Mol Syst Biol.

[37] Chang ST, Tchitchek N, Ghosh D, Benecke A, Katze MG (2011). A chemokine gene expression signature derived from meta-analysis predicts the pathogenicity of viral respiratory infections. BMC Syst Biol.

[38] Lukasiuk K, Dabrowski M, Adach A, Pitkanen A (2006). Epileptogenesis-related genes revisited. Prog Brain Res.

[39] van den Maagdenberg AMJM, Pizzorusso T, Kaja S, Terpolilli N, Shapovalova M, Hoebeek FE, Barrett CF, Gherardini L, van de Ven RC, Todorov B (2010). High cortical spreading depression susceptibility and migraine-associated symptoms in Ca(v)2.1 S218L mice. Ann Neurol.

[40] Vezzani A, French J, Bartfai T, Baram TZ (2011). The role of inflammation in epilepsy. Nat Rev Neurol.

[41] Vezzani A, Friedman A, Dingledine RJ (2013). The role of inflammation in epileptogenesis. Neuropharmacology.

[42] Nair A, Simonetti M, Birsa N, Ferrari MD, van den Maagdenberg AMJM, Giniatullin R, Nistri A, Fabbretti E (2010) Familial hemiplegic migraine Ca(v)2.1 channel mutation R192Q enhances ATP-gated P2X3 receptor activity of mouse sensory ganglion neurons mediating trigeminal pain. Mol Pain 6:48 10.1186/1744-8069-6-48 PMC2940876 20735819

[43] Ceruti S, Villa G, Fumagalli M, Colombo L, Magni G, Zanardelli M, Fabbretti E, Verderio C, van den Maagdenberg AMJM, Nistri A (2011). Calcitonin gene-related peptide-mediated enhancement of purinergic neuron/glia communication by the algogenic factor bradykinin in mouse trigeminal ganglia from wild-type and R192Q Cav2.1 knock-in mice: implications for basic mechanisms of migraine pain. J Neurosci.

[44] Franceschini A, Hullugundi SK, van den Maagdenberg AMJM, Nistri A, Fabbretti E (2013). Effects of LPS on P2X3 receptors of trigeminal sensory neurons and macrophages from mice expressing the R192Q Cacna1a gene mutation of familial hemiplegic migraine-1. Purinergic Signal.

[45] Franceschini A, Nair A, Bele T, van den Maagdenberg AMJM, Nistri A, Fabbretti E (2012). Functional crosstalk in culture between macrophages and trigeminal sensory neurons of a mouse genetic model of migraine. BMC Neurosci.

[46] Gursoy-Ozdemir Y, Qiu J, Matsuoka N, Bolay H, Bermpohl D, Jin H, Wang X, Rosenberg GA, Lo EH, Moskowitz MA (2004). Cortical spreading depression activates and upregulates MMP-9. J Clin Invest.

