# Supplementary material for: Cortical Spreading Depression Causes Unique Dysregulation of Inflammatory Pathways in a Transgenic Mouse Model of Migraine
Source: Mol Neurobiol. 2016 Mar 31;54(4):2986–96. doi: 10.1007/s12035-015-9681-5 (PMC5390001; doi:10.1007/s12035-015-9681-5)
Supplement: Supplementary file 1 — (PDF 454 kb) [file 12035_2015_9681_MOESM1_ESM.pdf]

Cortical Spreading Depression Causes Unique Dysregulation of Inflammatory Pathways in a Transgenic Mouse Model of Migraine

Journal: Molecular Neurobiology

Else Eising*, Reinald Shyti*, Peter A.C. ’t Hoen, Lisanne S. Vijfhuizen, Sjoerd M.H. Huisman, Ludo A. M. Broos, Ahmed Mahfouz, Marcel J.T. Reinders, Michel D. Ferrari, Else A. Tolner, Boukje de Vries, Arn M.J.M. van den Maagdenberg
Department of Human Genetics and Department of Neurology, Leiden University Medical Center, Leiden, The Netherlands. A.M.J.M.van_den_Maagdenberg@lumc.nl

Supplemental Figures

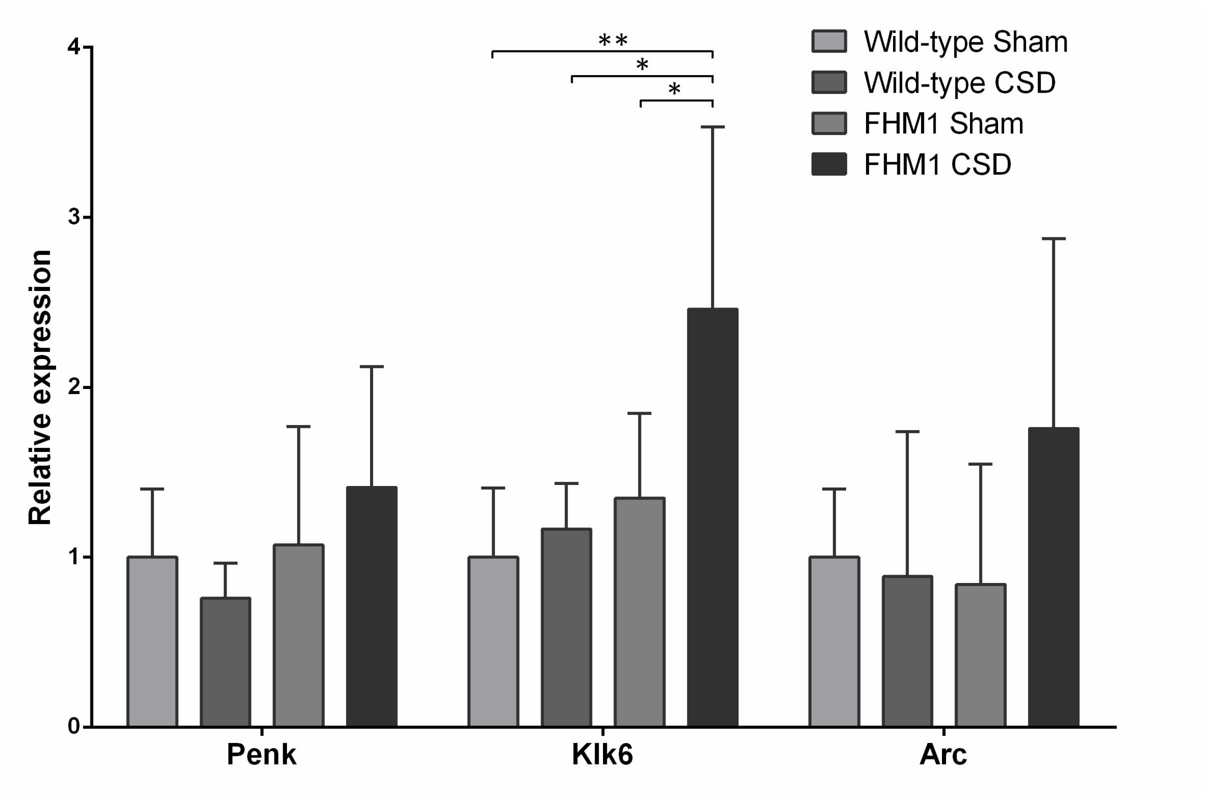

Supplemental Fig. 1 RT-qPCR validation of the ‘Genotype effect’ in biologically independent samples

Data were normalized to *Tbp* and *Gapdh* mRNA expression and expressed as fold-changes relative to the WT Sham-treated group (means ± SD). *p<0.05 and **p<0.01 according to a one-way ANOVA with Bonferroni post-hoc test.

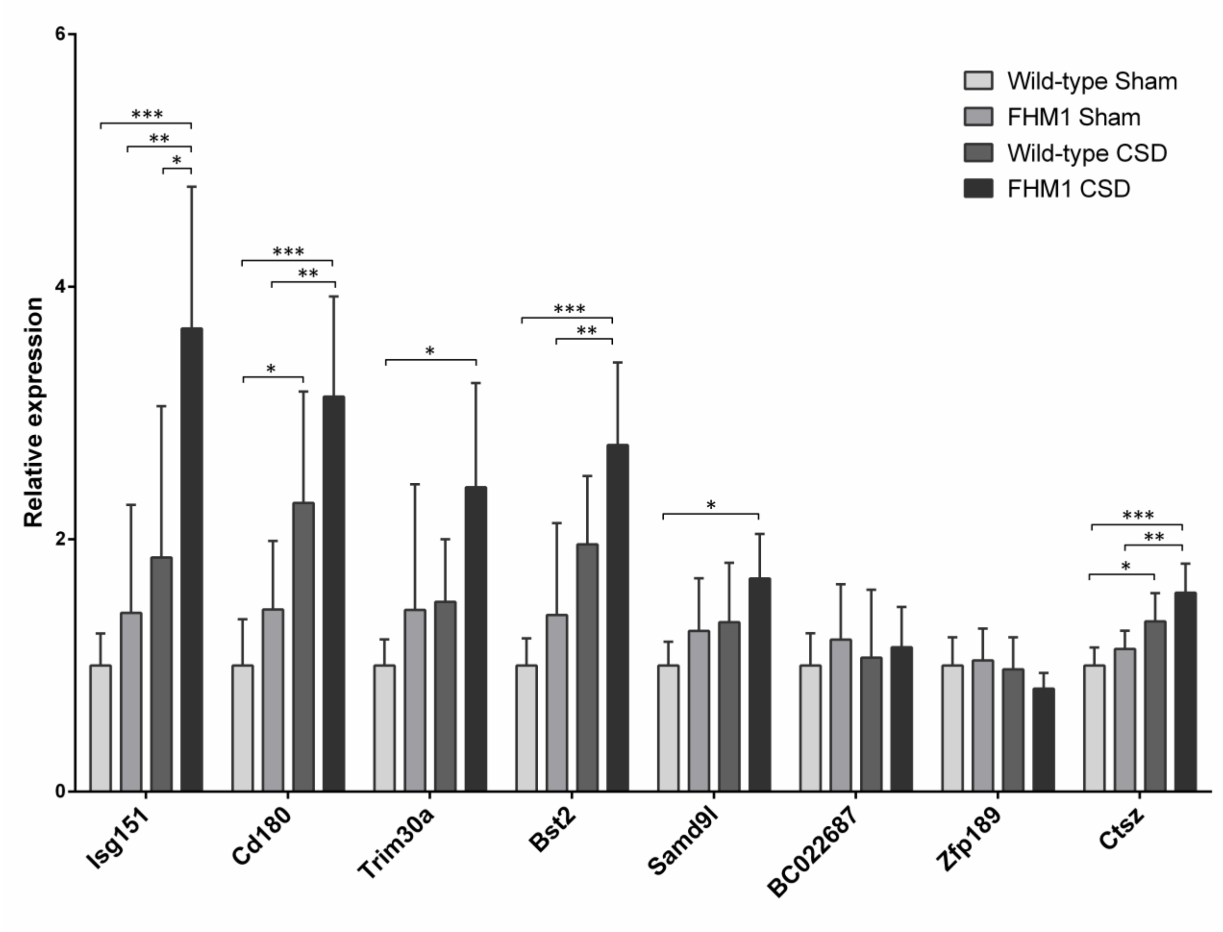

Supplemental Fig. 2: RT-qPCR validation of the 'CSD effect' in biologically independent samples. Data were normalized to *Tbp* and *Gapdh* mRNA expression and expressed as fold-changes relative to the WT Sham group (means ± SD). *p<0.05; **p<0.01 and ***p<0.001 according to a one-way ANOVA with Bonferroni post-hoc test.
