# Supplementary material for: Cortical Spreading Depression Causes Unique Dysregulation of Inflammatory Pathways in a Transgenic Mouse Model of Migraine
Source: Mol Neurobiol. 2016 Mar 31;54(4):2986–96. doi: 10.1007/s12035-015-9681-5 (PMC5390001; doi:10.1007/s12035-015-9681-5)
Supplement: Supplementary file 2 — (PDF 810 kb) [file 12035_2015_9681_MOESM2_ESM.pdf]

Cortical Spreading Depression Causes Unique Dysregulation of Inflammatory Pathways in a Transgenic Mouse Model of Migraine

Journal: Molecular Neurobiology

Else Eising*, Reinald Shyti*, Peter A.C. 't Hoen, Lisanne S. Vijfhuizen, Sjoerd M.H. Huisman, Ludo A. M. Broos, Ahmed Mahfouz, Marcel J.T. Reinders, Michel D. Ferrari, Else A. Tolner, Boukje de Vries, Arn M.J.M. van den Maagdenberg

Department of Human Genetics and Department of Neurology, Leiden University Medical Center, Leiden, The Netherlands. A.M.J.M.van_den_Maagdenberg@lumc.nl

Supplemental Tables

Supplemental Table 1: Primer sequences used for qPCR validation

Gene name	Forward primer (5' - 3')	Reverse primer (5' - 3')	Product size
Tbp	TCAAACCCAGAATTGTTCTCC	GTGGATAGGGAAGGCAGGA	168 bp
Gapdh	TGCACCACCAACTGCTTAGC	GGCATGGACTGTGGTCATGAG	87 bp
Penk	TCTGCAGCTCTTTTGCTTCA	TACTGGGAACGGGAGACAAC	147 bp
Klk6	CGATTCCTGTCAGGGTGATT	GGGGAGAACTGGATGTCTCA	194 bp
Arc	CCAAGAGGACCAAGGGTACA	GTGAAGACAAGCCAGCATGA	121 bp
Isg15	GAGCTAGAGCCTGCAGCAAT	TTCTGGGCAATCTGCTTCTT	122 bp
Cd180	GAGCCACCACATCCTCAGAT	TGCTGAAGGTCGTGTTTTGA	168 bp
Trim30a	GTTGGGGTTGAGCACACTTT	CCTCAGGGAAGCATTACTGG	90 bp
Bst2	CCTGGTTCAGCTTCGTGACT	GGAGTCCCTGGAGAAGAAGG	89 bp
Samd9l	GCTGCAAATCGGAAAGTCTC	CAGGGCCACTCAATCTCATT	118 bp
Bc022687	ATAAGGGGCAGAGCCAGTCT	GTTCCTGCAAGTGATCAGCA	139 bp
Zfp189	GGGAACTGACTTGGCGATTA	TGTGCCCTTCAGAACTTTCC	121 bp
Ctsz	ACTCTCGATGGCAAGGTTGT	GTGAGAAAGGCTGGATGAGG	80 bp
Spp1	TTCCAAAGAGAGCCAGGAGA	TTGTGGCTCTGATGTTCCAG	90 bp
Hmox1	CCCCAGATCAGCACTAGCTC	ATGGCATAAATTCCCACTGC	98 bp
Ifit1	GCCCAGATCTACCTGGACAA	CCTCACAGTCCATCTCAGCA	94 bp
Rsad2	CAATCCATCAAGAGGGCAGT	AAGGTCCCAGGTTTCGATCT	135 bp
Msn	CCCCAGAGCTAATTTGACCA	CAGAAGCAGGGCCTACTCAC	137 bp
Anxa2	GACCCTTTCCCCATCTTAGC	CCACTCCCTGGAATGTTCAC	86 bp
Nes	CTCGAGCAGGAAGTGGTAGG	GCCTCTTTTGGTTCCTTTCC	140 bp

Myd88	TGTCCCAAAGGAAACACACA	ACTGGCCTGAGCAACTAGGA	121 bp
Igtp	ATGTGTTTTGCCGTGAACAA	TGTGCCTCTGGTGTCTGAAG	100 bp
Hspa1a	GGCTGTCCTGCAAAACAAAT	ATTGCACGTGGGCTTTATCT	130 bp
Stat3	CCAGATTGCCCAAAGATAGC	CATGTGAGGAGCTGAAACCA	163 bp
Cd53	ATGCCAGTCAGGCAGGTAAT	CTGGGAATGTCCTTTGCACT	165 bp
Vim	AATGCTTCTCTGGCACGTCT	GCTCCTGGATCTCTTCATCG	100 bp
S1pr3	CAAGGCTCCCCATAAGTTGA	AGGGTTACCTAAGGCCCAGA	173 bp
Ifit3	GAGGACAACCGGAAGTGTGT	GGATGAGCAGAGGAGTCAGG	199 bp

Supplemental Table 2: **DeepSAGE sequencing depth and alignment results per sample**

**Sample name**	**Total number of reads**	**% uniquely mapped reads**	**% uniquely mapped reads aligned to exons**
WT Sham 1	11,149,652	69.74%	79.16%
WT Sham 2	15,694,763	70.06%	79.89%
WT Sham 3	22,066,897	70.04%	79.39%
WT Sham 4	28,140,164	64.07%	74.44%
WT Sham 5	28,204,032	69.48%	75.24%
WT Sham 6	34,929,188	70.80%	80.38%
WT CSD 1	26,526,992	66.31%	77.68%
WT CSD 2	18,184,816	70.84%	72.92%
WT CSD 3	18,211,472	71.47%	75.63%
WT CSD 4	18,340,236	71.91%	78.95%
WT CSD 5	38,281,251	66.59%	72.22%
WT CSD 6	53,091,348	66.12%	77.70%
FHM1 Sham 1	12,562,757	69.85%	78.56%
FHM1 Sham 2	14,625,022	71.14%	80.25%
FHM1 Sham 3	23,470,128	71.03%	75.41%
FHM1 Sham 4	28,557,774	68.62%	73.88%
FHM1 Sham 5	37,333,912	64.53%	74.23%
FHM1 Sham 6	41,027,465	65.61%	73.19%
FHM1 CSD 1	11,153,568	69.40%	74.60%
FHM1 CSD 2	13,932,460	70.18%	80.15%
FHM1 CSD 3	21,881,009	70.12%	74.90%
FHM1 CSD 4	23,267,739	69.39%	79.20%
FHM1 CSD 5	24,634,480	68.64%	79.14%
FHM1 CSD 6	42,624,969	62.35%	72.10%

Supplemental Table 3: Genes differentially expressed between genotypes

Ensembl gene ID	Gene name	Description	Chr[b]	p-value
ENSMUSG00000052566	*Hook2*	Hook homolog 2 (Drosophila)	8[a]	1.34×10^{-6}
ENSMUSG00000005161	*Prdx2*	Peroxiredoxin 2	8[a]	5.35×10^{-6}
ENSMUSG00000034729	*Mrps10*	Mitochondrial ribosomal protein S10	17	3.26×10^{-5}
ENSMUSG00000061723	*Tnnt3*	Troponin T3, skeletal, fast	7	6.56×10^{-5}
ENSMUSG00000056054	*S100a8*	S100 calcium binding protein A8 (calgranulin A)	3	6.63×10^{-5}
ENSMUSG00000030399	*Ckm*	Creatine kinase, muscle	7	1.07×10^{-4}
ENSMUSG00000000028	*Cdc45*	Cell division cycle 45 homolog (S. Cerevisiae)	16	1.24×10^{-4}
ENSMUSG00000003813	*Rad23a*	RAD23a homolog (S. Cerevisiae)	8[a]	1.60×10^{-4}
ENSMUSG00000033751	*Gadd45gip1*	Growth arrest and DNA-damage-inducible, gamma interacting protein 1	8[a]	2.09×10^{-4}
ENSMUSG00000085558	*4930412C18Rik*	RIKEN cdna 4930412C18 gene	4	2.96×10^{-4}
ENSMUSG00000031097	*Tnni2*	Troponin I, skeletal, fast 2	7	3.21×10^{-4}
ENSMUSG00000051747	*Ttn*	Titin	2	3.43×10^{-4}
ENSMUSG00000030730	*Atp2a1*	Atpase, Ca++ transporting, cardiac muscle, fast twitch 1	7	3.65×10^{-4}
ENSMUSG00000031698	*Mylk3*	Myosin light chain kinase 3	8[a]	4.14×10^{-4}
ENSMUSG00000031780	*Ccl17*	Chemokine (C-C motif) ligand 17	8[a]	4.30×10^{-4}
ENSMUSG00000054951	*9130008F23Rik*	RIKEN cdna 9130008F23 gene	17	6.06×10^{-4}
ENSMUSG00000055546	*Timd4*	T cell immunoglobulin and mucin domain containing 4	11	8.09×10^{-4}
ENSMUSG00000057003	*Myh4*	Myosin, heavy polypeptide 4, skeletal muscle	11	8.94×10^{-4}
ENSMUSG00000090551	*A730015C16Rik*	RIKEN cdna A730015C16 gene	4	9.18×10^{-4}
ENSMUSG00000090546	*Cdr1*	Cerebellar degeneration related antigen 1	X	1.00×10^{-3}
ENSMUSG00000047150	*1700001C19Rik*	RIKEN cdna 1700001C19 gene	17	1.14×10^{-3}
ENSMUSG00000085819	*A930028C08Rik*	RIKEN cdna A930028C08 gene	4	1.16×10^{-3}
ENSMUSG00000017300	*Tnnc2*	Troponin C2, fast	2	1.27×10^{-3}
ENSMUSG00000090244	*Gm16572*	Predicted gene 16572	12	1.34×10^{-3}
ENSMUSG00000033196	*Myh2*	Myosin, heavy polypeptide 2, skeletal muscle, adult	11	1.34×10^{-3}
ENSMUSG00000021622	*Ckmt2*	Creatine kinase, mitochondrial 2	13	1.45×10^{-3}
ENSMUSG00000006457	*Actn3*	Actinin alpha 3	19	1.46×10^{-3}
ENSMUSG00000031891	*Hsd11b2*	Hydroxysteroid 11-beta dehydrogenase 2	8[a]	1.54×10^{-3}
ENSMUSG00000045573	*Penk*	Preproenkephalin	4	1.66×10^{-3}
ENSMUSG00000040680	*Kremen2*	Kringle containing transmembrane protein 2	17	1.68×10^{-3}
ENSMUSG00000024989	*Cep55*	Centrosomal protein 55	19	1.77×10^{-3}
ENSMUSG00000085851	*4921518K17Rik*	RIKEN cdna 4921518K17 gene	12	1.83×10^{-3}
ENSMUSG00000030672	*Mylpf*	Myosin light chain, phosphorylatable, fast skeletal muscle	7	1.84×10^{-3}
ENSMUSG00000057074	*Ces1g*	Carboxylesterase 1G	8[a]	1.94×10^{-3}
ENSMUSG00000045903	*Npas4*	Neuronal PAS domain protein 4	19	2.17×10^{-3}
ENSMUSG00000050063	*Klk6*	Kallikrein related-peptidase 6	7	2.24×10^{-3}
ENSMUSG00000025317	*Car5a*	Carbonic anhydrase 5a, mitochondrial	8[a]	2.34×10^{-3}
ENSMUSG00000071036	*Gm10309*	Predicted gene 10309	17	2.43×10^{-3}
ENSMUSG00000046723	*Adam24*	A disintegrin and metallopeptidase domain 24 (testase 1)	8[a]	2.45×10^{-3}
ENSMUSG00000064918	*SNORD18*	Small nucleolar RNA SNORD18	9	2.47×10^{-3}
ENSMUSG00000072618	*Gm10384*	Predicted gene 10384	15	2.50×10^{-3}
ENSMUSG00000025473	*Adam8*	A disintegrin and metallopeptidase domain 8	7	2.58×10^{-3}
ENSMUSG00000056019	*Zfp709*	Zinc finger protein 709	8[a]	2.67×10^{-3}

ENSMUSG00000039133	*9330171B17Rik*	RIKEN cdna 9330171B17 gene	7	2.92×10^{-3}
ENSMUSG00000037991	*A630055G03Rik*	RIKEN cdna A630055G03 gene	16	3.17×10^{-3}
ENSMUSG00000086379	*1700026D11Rik*	RIKEN cdna 1700026D11 gene	2	3.29×10^{-3}
ENSMUSG00000068130	*Zfp442*	Zinc finger protein 442	2	3.39×10^{-3}
ENSMUSG00000024182	*Axin1*	Axin 1	17	3.55×10^{-3}
ENSMUSG00000046082	*Tmem174*	Transmembrane protein 174	13	3.58×10^{-3}
ENSMUSG00000087578	*Gm15605*	Predicted gene 15605	6	3.66×10^{-3}
ENSMUSG00000069922	*Ces3a*	Carboxylesterase 3A	8[a]	3.71×10^{-3}
ENSMUSG00000022602	*Arc*	Activity regulated cytoskeletal-associated protein	15	4.05×10^{-3}
ENSMUSG00000090291	*Lrrc10b*	Leucine rich repeat containing 10B	19	4.21×10^{-3}
ENSMUSG00000024078	*Ttc27*	Tetratricopeptide repeat domain 27	17	4.22×10^{-3}
ENSMUSG00000046844	*Vat1l*	Vesicle amine transport protein 1 homolog-like (T. Californica)	8[a]	4.36×10^{-3}
ENSMUSG00000022215	*Fitm1*	Fat storage-inducing transmembrane protein 1	14	4.40×10^{-3}
ENSMUSG00000035930	*Chst4*	Carbohydrate (chondroitin 6/keratan) sulfotransferase 4	8[a]	4.46×10^{-3}
ENSMUSG00000074828	*Gm10768*	Predicted gene 10768	19	4.75×10^{-3}
ENSMUSG00000085135	*Gm13713*	Predicted gene 13713	2	4.90×10^{-3}

[a]Differentially expressed genes that are located on chromosome 8; these genes are not included in GO-term and pathway analyses. [b]Chr: chromosome.

Supplemental Table 4: **Genes differentially expressed between Sham-treated and CSD-treated groups**

**Ensembl gene ID**	**Gene name**	**Description**	**Chr[a]**	**p-value**
ENSMUSG00000030156	*Cd69*	CD69 antigen	6	9.14×10^{-6}
ENSMUSG00000033355	*Rtp4*	Receptor transporter protein 4	16	2.48×10^{-5}
ENSMUSG00000035692	*Isg15*	ISG15 ubiquitin-like modifier	4	2.76×10^{-5}
ENSMUSG00000040026	*Saa3*	Serum amyloid A 3	7	3.38×10^{-5}
ENSMUSG00000083720	*Gm12901*	Predicted gene 12901	4	6.05×10^{-5}
ENSMUSG00000083487	*Gm13888*	Predicted gene 13888	2	8.61×10^{-5}
ENSMUSG00000052776	*Oas1a*	2'-5' oligoadenylate synthetase 1A	5	8.88×10^{-5}
ENSMUSG00000086905	*Gm13716*	Predicted gene 13716	2	9.10×10^{-5}
ENSMUSG00000081520	*Gm16200*	Predicted gene 16200	9	1.22×10^{-4}
ENSMUSG00000070871	*Ccnyl1*	Cyclin Y-like 1	1	1.71×10^{-4}
ENSMUSG00000086917	*Gm11630*	Predicted gene 11630	11	1.77×10^{-4}
ENSMUSG00000082410	*Gm12011*	Predicted gene 12011	11	1.84×10^{-4}
ENSMUSG00000060183	*Cxcl11*	Chemokine (C-X-C motif) ligand 11	5	2.13×10^{-4}
ENSMUSG00000036551	*Akap14*	A kinase (PRKA) anchor protein 14	X	2.14×10^{-4}
ENSMUSG00000088088		Rnase MRP	4	2.75×10^{-4}
ENSMUSG00000030107	*Usp18*	Ubiquitin specific peptidase 18	6	3.19×10^{-4}
ENSMUSG00000021624	*Cd180*	CD180 antigen	13	3.31×10^{-4}
ENSMUSG00000068606	*Gm4841*	Predicted gene 4841	18	3.34×10^{-4}
ENSMUSG00000065628	*Snord33*	Small nucleolar RNA, C/D box 33	7	3.88×10^{-4}
ENSMUSG00000062488	*I830012O16Rik*	RIKEN cdna I830012O16 gene	19	4.09×10^{-4}
ENSMUSG00000030921	*Trim30a*	Tripartite motif-containing 30A	7	4.66×10^{-4}
ENSMUSG00000031160	*Eras*	ES cell-expressed Ras	X	4.66×10^{-4}

ENSMUSG00000012211	*Tex22*	Testis expressed gene 22	12	5.74×10^{-4}
ENSMUSG00000068129	*Cst7*	Cystatin F (leukocystatin)	2	6.54×10^{-4}
ENSMUSG00000075279	*Mrpl23-ps1*	Mitichondrial ribosomal protein L23, pseudogene 1	2	6.59×10^{-4}
ENSMUSG00000015001	*Oc90*	Otoconin 90	15	6.89×10^{-4}
ENSMUSG00000039296	*Spdyb*	Speedy homolog B (Xenopus laevis)	5	7.06×10^{-4}
ENSMUSG00000000706	*Btn1a1*	Butyrophilin, subfamily 1, member A1	13	8.73×10^{-4}
ENSMUSG00000062235	*Gm5341*	Predicted pseudogene 5341	7	8.78×10^{-4}
ENSMUSG00000046718	*Bst2*	Bone marrow stromal cell antigen 2	8	9.58×10^{-4}
ENSMUSG00000001750	*Tcirg1*	T cell, immune regulator 1, atpase, H+ transporting, lysosomal V0 protein A3	19	9.81×10^{-4}
ENSMUSG00000081111	*Gm5913*	Predicted gene 5913	8	1.10×10^{-3}
ENSMUSG00000047735	*Samd9l*	Sterile alpha motif domain containing 9-like	6	1.21×10^{-3}
ENSMUSG00000007888	*Crlf1*	Cytokine receptor-like factor 1	8	1.33×10^{-3}
ENSMUSG00000034623	*Prss55*	Protease, serine, 55	14	1.35×10^{-3}
ENSMUSG00000055194	*Actbl2*	Actin, beta-like 2	13	1.40×10^{-3}
ENSMUSG00000086353	*Gm13481*	Predicted gene 13481	2	1.41×10^{-3}
ENSMUSG00000065110	*Snord61*	Small nucleolar RNA, C/D box 61	X	1.46×10^{-3}
ENSMUSG00000037594	*BC022687*	Cdna sequence BC022687	12	1.47×10^{-3}
ENSMUSG00000060989	*Gm11847*	Predicted gene 11847	4	1.48×10^{-3}
ENSMUSG00000046082	*Tmem174*	Transmembrane protein 174	13	1.50×10^{-3}
ENSMUSG00000028492	*Fam154a*	Family with sequence similarity 154, member A	4	1.57×10^{-3}
ENSMUSG00000079488	*Cd47*	CD47 antigen (Rh-related antigen, integrin-associated signal transducer)	16	1.68×10^{-3}
ENSMUSG00000084835	*Gm12352*	Predicted gene 12352	11	1.68×10^{-3}
ENSMUSG00000082429	*Gm13171*	Predicted gene 13171	4	1.74×10^{-3}
ENSMUSG00000064904		U6 spliceosomal RNA	3	1.77×10^{-3}
ENSMUSG00000025577	*Cbx2*	Chromobox homolog 2 (Drosophila Pc class)	11	1.90×10^{-3}
ENSMUSG00000054404	*Slfn5*	Schlafen 5	11	1.91×10^{-3}
ENSMUSG00000087589	*D430040D24Rik*	RIKEN cdna D430040D24 gene	1	1.95×10^{-3}
ENSMUSG00000034855	*Cxcl10*	Chemokine (C-X-C motif) ligand 10	5	2.03×10^{-3}
ENSMUSG00000030823	*9130019O22Rik*	RIKEN cdna 9130019O22 gene	7	2.06×10^{-3}
ENSMUSG00000020534	*Shmt1*	Serine hydroxymethyltransferase 1 (soluble)	11	2.13×10^{-3}
ENSMUSG00000074203	*G430095P16Rik*	RIKEN cdna G430095P16 gene	8	2.31×10^{-3}
ENSMUSG00000089829	*Gm16565*	Predicted gene 16565	1	2.36×10^{-3}
ENSMUSG00000062270	*Morf4l1*	Mortality factor 4 like 1	9	2.46×10^{-3}
ENSMUSG00000087342	*Gm12238*	Predicted gene 12238	11	2.46×10^{-3}
ENSMUSG00000065061		Small nucleolar RNA SNORA29	11	2.49×10^{-3}
ENSMUSG00000039634	*Zfp189*	Zinc finger protein 189	4	2.69×10^{-3}
ENSMUSG00000027379	*Bub1*	Budding uninhibited by benzimidazoles 1 homolog (S. Cerevisiae)	2	2.84×10^{-3}
ENSMUSG00000030562	*Nox4*	NADPH oxidase 4	7	2.91×10^{-3}
ENSMUSG00000086773	*Gm16192*	Predicted gene 16192	8	2.92×10^{-3}
ENSMUSG00000050468	*Astl*	Astacin-like metalloendopeptidase (M12 family)	2	2.96×10^{-3}
ENSMUSG00000021748	*Pdhb*	Pyruvate dehydrogenase (lipoamide) beta	14	3.05×10^{-3}
ENSMUSG00000090840	*1700092M07Rik*	RIKEN cdna 1700092M07 gene	19	3.20×10^{-3}
ENSMUSG00000034641	*Cd300ld*	CD300 molecule-like family member d	11	3.30×10^{-3}
ENSMUSG00000036770	*4933433C11Rik*	RIKEN cdna 4933433C11 gene	2	3.36×10^{-3}
ENSMUSG00000038147	*Cd84*	CD84 antigen	1	3.40×10^{-3}
ENSMUSG00000085531	*Gm12233*	Predicted gene 12233	11	3.54×10^{-3}

ENSMUSG00000041827	*Oasl1*	2'-5' oligoadenylate synthetase-like 1	5	3.58×10^{-3}
ENSMUSG00000037499	*Nenf*	Neuron derived neurotrophic factor	1	3.59×10^{-3}
ENSMUSG00000031461	*Myom2*	Myomesin 2	8	3.72×10^{-3}
ENSMUSG00000074412	*Gm10689*	Predicted gene 10689	8	3.73×10^{-3}
ENSMUSG00000085203	*Gm12927*	Predicted gene 12927	4	3.74×10^{-3}
ENSMUSG00000026073	*Il1r2*	Interleukin 1 receptor, type II	1	3.74×10^{-3}
ENSMUSG00000076230			2	4.00×10^{-3}
ENSMUSG00000061272	*Gm14173*	Predicted gene 14173	2	4.02×10^{-3}
ENSMUSG00000016256	*Ctsz*	Cathepsin Z	2	4.12×10^{-3}
ENSMUSG00000031255	*Sytl4*	Synaptotagmin-like 4	X	4.33×10^{-3}
ENSMUSG00000062381	*Vps28*	Vacuolar protein sorting 28 (yeast)	15	4.33×10^{-3}
ENSMUSG00000063656	*Gm10135*	Predicted gene 10135	5	4.44×10^{-3}

[a]Chr: chromosome.

Supplemental Table 5: Genes differentially expressed due to the interaction of genotype and CSD effect

Ensembl gene ID	Gene name	Description	Chr[a]	Cluster[b]	P-value
ENSMUSG00000029664	*Tfpi2*	Tissue factor pathway inhibitor 2	6	1	1.51×10^{-8}
ENSMUSG00000071637	*Cebpd*	CCAAT/enhancer binding protein (C/EBP), delta	16	1	2.13×10^{-6}
ENSMUSG00000063193	*Cd300lb*	CD300 antigen like family member B	11	1	3.28×10^{-6}
ENSMUSG00000029304	*Spp1*	Secreted phosphoprotein 1	5	1	3.48×10^{-6}
ENSMUSG00000070327	*Rnf213*	Ring finger protein 213	11	1	6.47×10^{-6}
ENSMUSG00000005413	*Hmox1*	Heme oxygenase (decycling) 1	8	1	8.13×10^{-6}
ENSMUSG00000020676	*Ccl11*	Chemokine (C-C motif) ligand 11	11	1	1.57×10^{-5}
ENSMUSG00000019987	*Arg1*	Arginase, liver	10	1	2.22×10^{-5}
ENSMUSG00000074896	*Ifit3*	Interferon-induced protein with tetratricopeptide repeats 3	19	1	2.45×10^{-5}
ENSMUSG00000026535	*Ifi202b*	Interferon activated gene 202B	1	1	3.51×10^{-5}
ENSMUSG00000035493	*Tgfbi*	Transforming growth factor, beta induced	13	1	3.53×10^{-5}
ENSMUSG00000024679	*Ms4a6d*	Membrane-spanning 4-domains, subfamily A, member 6D	19	1	4.09×10^{-5}
ENSMUSG00000068245	*D14Ertd668e*	DNA segment, Chr 14, ERATO Doi 668, expressed	14	1	4.41×10^{-5}
ENSMUSG00000022876	*Samsn1*	SAM domain, SH3 domain and nuclear localization signals, 1	16	1	5.50×10^{-5}
ENSMUSG00000030748	*Il4ra*	Interleukin 4 receptor, alpha	7	1	8.27×10^{-5}
ENSMUSG00000025044	*Msr1*	Macrophage scavenger receptor 1	8	1	8.95×10^{-5}
ENSMUSG00000004709	*Cd244*	CD244 natural killer cell receptor 2B4	1	1	1.19×10^{-4}
ENSMUSG00000027800	*Tm4sf1*	Transmembrane 4 superfamily member 1	3	1	1.24×10^{-4}
ENSMUSG00000040552	*C3ar1*	Complement component 3a receptor 1	6	1	1.34×10^{-4}
ENSMUSG00000020592	*Sdc1*	Syndecan 1	12	1	1.40×10^{-4}
ENSMUSG00000025498	*Irf7*	Interferon regulatory factor 7	7	1	1.45×10^{-4}
ENSMUSG00000026580	*Selp*	Selectin, platelet	1	1	1.52×10^{-4}
ENSMUSG00000031207	*Msn*	Moesin	X	1	1.57×10^{-4}
ENSMUSG00000032231	*Anxa2*	Annexin A2	9	1	1.58×10^{-4}
ENSMUSG00000048572	*E030010A14Rik*	RIKEN cdna E030010A14 gene	19	1	1.76×10^{-4}
ENSMUSG00000022150	*Dab2*	Disabled homolog 2 (Drosophila)	15	1	1.82×10^{-4}
ENSMUSG00000018920	*Cxcl16*	Chemokine (C-X-C motif) ligand 16	11	1	1.87×10^{-4}

ENSMUSG00000018930	*Ccl4*	Chemokine (C-C motif) ligand 4	11	1	2.31×10^{-4}
ENSMUSG00000055632	*Hmcn2*	Hemicentin 2	2	1	2.53×10^{-4}
ENSMUSG00000004891	*Nes*	Nestin	3	1	2.97×10^{-4}
ENSMUSG00000029552	*Tes*	Testis derived transcript	6	1	3.54×10^{-4}
ENSMUSG00000049130	*C5ar1*	Complement component 5a receptor 1	7	1	3.88×10^{-4}
ENSMUSG00000023914	*Mep1a*	Meprin 1 alpha	17	1	5.01×10^{-4}
ENSMUSG00000028480	*Glipr2*	GLI pathogenesis-related 2	4	1	5.08×10^{-4}
ENSMUSG00000001131	*Timp1*	Tissue inhibitor of metalloproteinase 1	X	1	5.67×10^{-4}
ENSMUSG00000032508	*Myd88*	Myeloid differentiation primary response gene 88	9	1	6.46×10^{-4}
ENSMUSG00000078853	*Igtp*	Interferon gamma induced gtpase	11	1	6.49×10^{-4}
ENSMUSG00000024590	*Lmnb1*	Lamin B1	18	1	6.52×10^{-4}
ENSMUSG00000035385	*Ccl2*	Chemokine (C-C motif) ligand 2	11	1	7.23×10^{-4}
ENSMUSG00000028268	*Gbp3*	Guanylate binding protein 3	3	1	8.65×10^{-4}
ENSMUSG00000000263	*Glra1*	Glycine receptor, alpha 1 subunit	11	1	9.27×10^{-4}
ENSMUSG00000014813	*Stc1*	Stanniocalcin 1	14	1	1.09×10^{-3}
ENSMUSG00000044957	*4921523A10Rik*	RIKEN cdna 4921523A10 gene	17	1	1.12×10^{-3}
ENSMUSG00000004040	*Stat3*	Signal transducer and activator of transcription 3	11	1	1.15×10^{-3}
ENSMUSG00000091649	*Gm4902*	Predicted gene 4902	14	1	1.42×10^{-3}
ENSMUSG00000050578	*Mmp13*	Matrix metallopeptidase 13	9	1	1.44×10^{-3}
ENSMUSG00000042608	*Stk40*	Serine/threonine kinase 40	4	1	1.51×10^{-3}
ENSMUSG00000016524	*Il19*	Interleukin 19	1	1	1.64×10^{-3}
ENSMUSG00000040747	*Cd53*	CD53 antigen	3	1	1.76×10^{-3}
ENSMUSG00000050370	*Ch25h*	Cholesterol 25-hydroxylase	19	1	1.84×10^{-3}
ENSMUSG00000000555	*Itga5*	Integrin alpha 5 (fibronectin receptor alpha)	15	1	1.85×10^{-3}
ENSMUSG00000073455	*Gm3435*	Predicted gene 3435	17	1	1.96×10^{-3}
ENSMUSG00000028037	*Ifi44*	Interferon-induced protein 44	3	1	2.06×10^{-3}
ENSMUSG00000030560	*Ctsc*	Cathepsin C	7	1	2.06×10^{-3}
ENSMUSG00000026728	*Vim*	Vimentin	2	1	2.08×10^{-3}
ENSMUSG00000037411	*Serpine1*	Serine (or cysteine) peptidase inhibitor, clade E, member 1	5	1	2.10×10^{-3}
ENSMUSG00000053113	*Socs3*	Suppressor of cytokine signaling 3	11	1	2.13×10^{-3}
ENSMUSG00000026832	*Cytip*	Cytohesin 1 interacting protein	2	1	2.16×10^{-3}
ENSMUSG00000053063	*Clec12a*	C-type lectin domain family 12, member a	6	1	2.19×10^{-3}
ENSMUSG00000032501	*Trib1*	Tribbles homolog 1 (Drosophila)	15	1	2.30×10^{-3}
ENSMUSG00000044678	*Ly6k*	Lymphocyte antigen 6 complex, locus K	15	1	2.32×10^{-3}
ENSMUSG00000067586	*S1pr3*	Sphingosine-1-phosphate receptor 3	13	1	2.35×10^{-3}
ENSMUSG00000029561	*Oasl2*	2'-5' oligoadenylate synthetase-like 2	5	1	2.39×10^{-3}
ENSMUSG00000062345	*Serpinb2*	Serine (or cysteine) peptidase inhibitor, clade B, member 2	1	1	2.70×10^{-3}
ENSMUSG00000091971	*Hspa1a*	Heat shock protein 1A	17	1	2.73×10^{-3}
ENSMUSG00000035929	*H2-Q4*	Histocompatibility 2, Q region locus 4	17	1	2.79×10^{-3}
ENSMUSG00000040152	*Thbs1*	Thrombospondin 1	2	1	2.98×10^{-3}
ENSMUSG00000070533	*Wfdc8*	WAP four-disulfide core domain 8	2	1	2.99×10^{-3}
ENSMUSG00000031722	*Hp*	Haptoglobin	8	1	3.00×10^{-3}
ENSMUSG00000034459	*Ifit1*	Interferon-induced protein with tetratricopeptide repeats 1	19	1	3.09×10^{-3}
ENSMUSG00000020641	*Rsad2*	Radical S-adenosyl methionine domain containing 2	12	1	3.13×10^{-3}
ENSMUSG00000042190	*Cmklr1*	Chemokine-like receptor 1	5	1	3.16×10^{-3}
ENSMUSG00000039853	*Trim14*	Tripartite motif-containing 14	4	1	3.22×10^{-3}

ENSMUSG00000023249	*Parp3*	Poly (ADP-ribose) polymerase family, member 3	9	1	3.22×10^{-3}
ENSMUSG00000079227	*Ccr5*	Chemokine (C-C motif) receptor 5	9	1	3.26×10^{-3}
ENSMUSG00000068699	*Flnc*	Filamin C, gamma	6	1	3.41×10^{-3}
ENSMUSG00000022367	*Has2*	Hyaluronan synthase 2	15	1	3.41×10^{-3}
ENSMUSG00000024907	*Gal*	Galanin	19	1	3.76×10^{-3}
ENSMUSG00000003541	*Ier3*	Immediate early response 3	17	1	3.76×10^{-3}
ENSMUSG00000013091	*Tmem190*	Transmembrane protein 190	7	1	3.84×10^{-3}
ENSMUSG00000029163	*Emilin1*	Elastin microfibril interfacer 1	5	1	3.88×10^{-3}
ENSMUSG00000020427	*Igfbp3*	Insulin-like growth factor binding protein 3	11	1	3.94×10^{-3}
ENSMUSG00000029423	*Piwil1*	Piwi-like homolog 1 (Drosophila)	5	1	4.02×10^{-3}
ENSMUSG00000004952	*Rasa4*	RAS p21 protein activator 4	5	1	4.09×10^{-3}
ENSMUSG00000063594	*Gng8*	Guanine nucleotide binding protein (G protein), gamma 8	7	1	4.24×10^{-3}
ENSMUSG00000030745	*Il21r*	Interleukin 21 receptor	7	1	4.26×10^{-3}
ENSMUSG00000054988	*Agtr1b*	Angiotensin II receptor, type 1b	3	1	4.37×10^{-3}
ENSMUSG00000050410	*Tcf19*	Transcription factor 19	17	1	4.47×10^{-3}
ENSMUSG00000035105	*Egln3*	EGL nine homolog 3 (C. Elegans)	12	1	4.57×10^{-3}
ENSMUSG00000051048	*P4ha3*	Procollagen-proline, 2-oxoglutarate 4-dioxygenase (proline 4-hydroxylase), alpha polypeptide III	7	1	4.54×10^{-3}
ENSMUSG00000026749	*Nek6*	NIMA (never in mitosis gene a)-related expressed kinase 6	2	1	4.63×10^{-3}
ENSMUSG00000040663	*Clcf1*	Cardiotrophin-like cytokine factor 1	19	1	4.66×10^{-3}
ENSMUSG00000037095	*Lrg1*	Leucine-rich alpha-2-glycoprotein 1	17	1	4.68×10^{-3}
ENSMUSG00000091204	*Gm7271*	Predicted gene 7271	5	1	4.69×10^{-3}
ENSMUSG00000036256	*Igfbp7*	Insulin-like growth factor binding protein 7	5	1	4.78×10^{-3}
ENSMUSG00000047250	*Ptgs1*	Prostaglandin-endoperoxide synthase 1	2	2	3.07×10^{-6}
ENSMUSG00000044258	*Ctla2a*	Cytotoxic T lymphocyte-associated protein 2 alpha	13	2	4.99×10^{-6}
ENSMUSG00000000628	*Hk2*	Hexokinase 2	6	2	9.16×10^{-6}
ENSMUSG00000031762	*Mt2*	Metallothionein 2	8	2	3.31×10^{-5}
ENSMUSG00000046321	*Hs3st2*	Heparan sulfate (glucosamine) 3-O-sulfotransferase 2	7	2	4.27×10^{-5}
ENSMUSG00000021365	*Nedd9*	Neural precursor cell expressed, developmentally down-regulated gene 9	13	2	4.53×10^{-5}
ENSMUSG00000046768	*Rhoj*	Ras homolog gene family, member J	12	2	4.98×10^{-5}
ENSMUSG00000057346	*Apol9b*	Apolipoprotein L 9b	15	2	6.03×10^{-5}
ENSMUSG00000062609	*Kcnj15*	Potassium inwardly-rectifying channel, subfamily J, member 15	16	2	7.73×10^{-5}
ENSMUSG00000090394	*4930523C07Rik*	RIKEN cdna 4930523C07 gene	1	2	9.99×10^{-5}
ENSMUSG00000090958	*Lrrc32*	Leucine rich repeat containing 32	7	2	1.04×10^{-4}
ENSMUSG00000024486	*Hbegf*	Heparin-binding EGF-like growth factor	18	2	1.21×10^{-4}
ENSMUSG00000047407	*Tgif1*	TGFB-induced factor homeobox 1	17	2	1.27×10^{-4}
ENSMUSG00000030022	*Adamts9*	A disintegrin-like and metallopeptidase (reprolysin type) with thrombospondin type 1 motif, 9	6	2	1.38×10^{-4}
ENSMUSG00000028464	*Tpm2*	Tropomyosin 2, beta	4	2	2.35×10^{-4}
ENSMUSG00000020388	*Pdlim4*	PDZ and LIM domain 4	11	2	2.88×10^{-4}
ENSMUSG00000039956	*Mrap*	Melanocortin 2 receptor accessory protein	16	2	3.22×10^{-4}
ENSMUSG00000022500	*Litaf*	LPS-induced TN factor	16	2	3.45×10^{-4}
ENSMUSG00000044701	*Il27*	Interleukin 27	7	2	3.55×10^{-4}
ENSMUSG00000063232	*Serpina11*	Serine (or cysteine) peptidase inhibitor,	12	2	4.11×10^{-4}

		clade A (alpha-1 antiproteinase, antitrypsin), member 11			
ENSMUSG00000051159	*Cited1*	Cbp/p300-interacting transactivator with Glu/Asp-rich carboxy-terminal domain 1	X	2	4.13×10^{-4}
ENSMUSG00000078934	*9230113P08Rik*	RIKEN cdna 9230113P08 gene	9	2	4.28×10^{-4}
ENSMUSG00000085712	*Gm15124*	Predicted gene 15124	10	2	4.94×10^{-4}
ENSMUSG00000079445	*B3gnt7*	UDP-glcnac:betagal beta-1,3-N-acetylglucosaminyltransferase 7	1	2	4.99×10^{-4}
ENSMUSG00000027261	*Hao1*	Hydroxyacid oxidase 1, liver	2	2	5.80×10^{-4}
ENSMUSG00000060962	*Dmkn*	Dermokine	7	2	6.16×10^{-4}
ENSMUSG00000072812	*Ahnak2*	AHNAK nucleoprotein 2	12	2	6.22×10^{-4}
ENSMUSG00000001473	*Tubb6*	Tubulin, beta 6 class V	18	2	7.03×10^{-4}
ENSMUSG00000020017	*Hal*	Histidine ammonia lyase	10	2	7.58×10^{-4}
ENSMUSG00000026271	*Gpr35*	G protein-coupled receptor 35	1	2	7.58×10^{-4}
ENSMUSG00000039899	*Fgl2*	Fibrinogen-like protein 2	5	2	7.64×10^{-4}
ENSMUSG00000032024	*9030425E11Rik*	RIKEN cdna 9030425E11 gene	9	2	7.84×10^{-4}
ENSMUSG00000022094	*Slc39a14*	Solute carrier family 39 (zinc transporter), member 14	14	2	8.30×10^{-4}
ENSMUSG00000090698	*Apold1*	Apolipoprotein L domain containing 1	6	2	9.25×10^{-4}
ENSMUSG00000087596	*Gm11715*	Predicted gene 11715	11	2	9.31×10^{-4}
ENSMUSG00000024749	*Tmc1*	Transmembrane channel-like gene family 1	19	2	9.81×10^{-4}
ENSMUSG00000022015	*Tnfsf11*	Tumor necrosis factor (ligand) superfamily, member 11	14	2	1.01×10^{-3}
ENSMUSG00000027435	*Cd93*	CD93 antigen	2	2	1.05×10^{-3}
ENSMUSG00000054215	*Sprr2k*	Small proline-rich protein 2K	3	2	1.15×10^{-3}
ENSMUSG00000023067	*Cdkn1a*	Cyclin-dependent kinase inhibitor 1A (P21)	17	2	1.17×10^{-3}
ENSMUSG00000056498	*Tmem154*	Transmembrane protein 154	3	2	1.25×10^{-3}
ENSMUSG00000057534	*Gm15698*	Predicted gene 15698	11	2	1.29×10^{-3}
ENSMUSG00000031825	*Crispld2*	Cysteine-rich secretory protein LCCL domain containing 2	8	2	1.30×10^{-3}
ENSMUSG00000053797	*Krt16*	Keratin 16	11	2	1.38×10^{-3}
ENSMUSG00000022528	*Hes1*	Hairy and enhancer of split 1 (Drosophila)	16	2	1.39×10^{-3}
ENSMUSG00000019846	*Lama4*	Laminin, alpha 4	10	2	1.54×10^{-3}
ENSMUSG00000057596	*Trim30d*	Tripartite motif-containing 30D	7	2	1.55×10^{-3}
ENSMUSG00000006411	*Pvrl4*	Poliovirus receptor-related 4	1	2	1.56×10^{-3}
ENSMUSG00000001168	*Oas1h*	2'-5' oligoadenylate synthetase 1H	5	2	1.71×10^{-3}
ENSMUSG00000020911	*Krt19*	Keratin 19	11	2	1.77×10^{-3}
ENSMUSG00000017009	*Sdc4*	Syndecan 4	2	2	1.79×10^{-3}
ENSMUSG00000085603	*Gm11346*	Predicted gene 11346	13	2	1.79×10^{-3}
ENSMUSG00000034185	*6430628N08Rik*	RIKEN cdna 6430628N08 gene	4	2	1.80×10^{-3}
ENSMUSG00000002233	*Rhoc*	Ras homolog gene family, member C	3	2	1.83×10^{-3}
ENSMUSG00000006546	*Cryba2*	Crystallin, beta A2	1	2	1.92×10^{-3}
ENSMUSG00000040711	*Sh3pxd2b*	SH3 and PX domains 2B	11	2	1.99×10^{-3}
ENSMUSG00000023341	*Mx2*	Myxovirus (influenza virus) resistance 2	16	2	2.02×10^{-3}
ENSMUSG00000000359	*Rem1*	Rad and gem related GTP binding protein 1	2	2	2.17×10^{-3}
ENSMUSG00000057278	*Snrpg*	Small nuclear ribonucleoprotein polypeptide G	6	2	2.24×10^{-3}
ENSMUSG00000049719	*Prss46*	Protease, serine, 46	9	2	2.27×10^{-3}
ENSMUSG00000066553	*Gm6969*	Predicted pseudogene 6969	12	2	2.28×10^{-3}
ENSMUSG00000049892	*Rasd1*	RAS, dexamethasone-induced 1	11	2	2.30×10^{-3}
ENSMUSG00000086229	*Gm4887*	Predicted gene 4887	7	2	2.40×10^{-3}
ENSMUSG00000027762	*Sucnr1*	Succinate receptor 1	3	2	2.43×10^{-3}

ENSMUSG00000002228	*Ppm1j*	Protein phosphatase 1J	3	2	2.47×10^{-3}
ENSMUSG00000055312	*0610012H03Rik*	RIKEN cdna 0610012H03 gene	2	2	2.55×10^{-3}
ENSMUSG00000055301	*Adh7*	Alcohol dehydrogenase 7 (class IV), mu or sigma polypeptide	3	2	2.67×10^{-3}
ENSMUSG00000001023	*S100a5*	S100 calcium binding protein A5	3	2	2.69×10^{-3}
ENSMUSG00000023015	*Racgap1*	Rac gtpase-activating protein 1	15	2	2.74×10^{-3}
ENSMUSG00000050201	*Otop2*	Otopetrin 2	11	2	2.81×10^{-3}
ENSMUSG00000028978	*Nos3*	Nitric oxide synthase 3, endothelial cell	5	2	2.82×10^{-3}
ENSMUSG00000053475	*Tnfaip6*	Tumor necrosis factor alpha induced protein 6	2	2	2.90×10^{-3}
ENSMUSG00000038007	*Acer2*	Alkaline ceramidase 2	4	2	2.91×10^{-3}
ENSMUSG00000053175	*Bcl3*	B cell leukemia/lymphoma 3	7	2	2.92×10^{-3}
ENSMUSG00000048721	*C030019I05Rik*	RIKEN cdna C030019I05 gene	11	2	2.95×10^{-3}
ENSMUSG00000030110	*Ret*	Ret proto-oncogene	6	2	3.30×10^{-3}
ENSMUSG00000071104	*Ccdc110*	Coiled-coil domain containing 110	8	2	3.34×10^{-3}
ENSMUSG00000087175	*Gm15133*	Predicted gene 15133	7	2	3.50×10^{-3}
ENSMUSG00000028738	*Tas1r2*	Taste receptor, type 1, member 2	4	2	3.54×10^{-3}
ENSMUSG00000061531	*Tmem236*	Transmembrane protein 236	2	2	3.66×10^{-3}
ENSMUSG00000032014	*Oaf*	OAF homolog (Drosophila)	9	2	3.67×10^{-3}
ENSMUSG00000035678	*Tnfsf9*	Tumor necrosis factor (ligand) superfamily, member 9	17	2	3.75×10^{-3}
ENSMUSG00000022479	*Vdr*	Vitamin D receptor	15	2	3.86×10^{-3}
ENSMUSG00000032802	*Srxn1*	Sulfiredoxin 1 homolog (S. Cerevisiae)	2	2	3.87×10^{-3}
ENSMUSG00000074743	*Thbd*	Thrombomodulin	2	2	3.96×10^{-3}
ENSMUSG00000082651	*Gm14001*	Predicted gene 14001	2	2	3.96×10^{-3}
ENSMUSG00000063652	*Slc22a21*	Solute carrier family 22 (organic cation transporter), member 21	11	2	4.04×10^{-3}
ENSMUSG00000070717	*Gm10300*	Predicted gene 10300	4	2	4.11×10^{-3}
ENSMUSG00000030607	*Acan*	Aggrecan	7	2	4.28×10^{-3}
ENSMUSG00000024678	*Ms4a4d*	Membrane-spanning 4-domains, subfamily A, member 4D	19	2	4.35×10^{-3}
ENSMUSG00000043505	*Gimap5*	Gtpase, IMAP family member 5	6	2	4.37×10^{-3}
ENSMUSG00000016526	*Dyrk3*	Dual-specificity tyrosine-(Y)-phosphorylation regulated kinase 3	1	2	4.43×10^{-3}
ENSMUSG00000026836	*Acvr1*	Activin A receptor, type 1	2	2	4.44×10^{-3}
ENSMUSG00000021499	*Catsper3*	Cation channel, sperm associated 3	13	2	4.44×10^{-3}
ENSMUSG00000018143	*Mafk*	V-maf musculoaponeurotic fibrosarcoma oncogene family, protein K (avian)	5	2	4.46×10^{-3}
ENSMUSG00000086342	*Gm12932*	Predicted gene 12932	4	2	4.46×10^{-3}
ENSMUSG00000064768		Small nucleolar RNA SNORD60	17	2	4.59×10^{-3}
ENSMUSG00000028063	*Lmna*	Lamin A	3	2	4.68×10^{-3}
ENSMUSG00000033022	*Cdo1*	Cysteine dioxygenase 1, cytosolic	18	2	4.83×10^{-3}
ENSMUSG00000083563	*Gm13340*	Predicted gene 13340	2	2	4.86×10^{-3}
ENSMUSG00000069713	*4933406P04Rik*	RIKEN cdna 4933406P04 gene	10	2	4.95×10^{-3}
ENSMUSG00000070891	*Gm12689*	Predicted gene 12689	4	3	5.64×10^{-6}
ENSMUSG00000050619	*Zscan29*	Zinc finger SCAN domains 29	2	3	4.51×10^{-5}
ENSMUSG00000035033	*Tbr1*	T-box brain gene 1	2	3	5.02×10^{-5}
ENSMUSG00000058626	*Capn11*	Calpain 11	17	3	8.43×10^{-5}
ENSMUSG00000033715	*Akr1c14*	Aldo-keto reductase family 1, member C14	13	3	1.14×10^{-4}
ENSMUSG00000027570	*Col9a3*	Collagen, type IX, alpha 3	2	3	1.19×10^{-4}
ENSMUSG00000027239	*Mdk*	Midkine	2	3	1.77×10^{-4}
ENSMUSG00000022684	*Bfar*	Bifunctional apoptosis regulator	16	3	1.82×10^{-4}

ENSMUSG00000052229	*Gpr17*	G protein-coupled receptor 17	18	3	2.27×10^{-4}
ENSMUSG00000028919	*Arhgef19*	Rho guanine nucleotide exchange factor (GEF) 19	4	3	2.44×10^{-4}
ENSMUSG00000040054	*Baz2a*	Bromodomain adjacent to zinc finger domain, 2A	10	3	4.32×10^{-4}
ENSMUSG00000034509	*Mad2l1bp*	MAD2L1 binding protein	17	3	4.69×10^{-4}
ENSMUSG00000044986	*Tst*	Thiosulfate sulfurtransferase, mitochondrial	15	3	5.83×10^{-4}
ENSMUSG00000035805	*Mlc1*	Megalencephalic leukoencephalopathy with subcortical cysts 1 homolog (human)	15	3	8.86×10^{-4}
ENSMUSG00000027646	*Src*	Rous sarcoma oncogene	2	3	8.99×10^{-4}
ENSMUSG00000089707	*4921525B02Rik*	RIKEN cdna 4921525B02 gene	14	3	9.09×10^{-4}
ENSMUSG00000033032	*Afap1l1*	Actin filament associated protein 1-like 1	18	3	9.61×10^{-4}
ENSMUSG00000031137	*Fgf13*	Fibroblast growth factor 13	X	3	9.81×10^{-4}
ENSMUSG00000029335	*Bmp3*	Bone morphogenetic protein 3	5	3	9.94×10^{-4}
ENSMUSG00000007207	*Stx1a*	Syntaxin 1A (brain)	5	3	9.97×10^{-4}
ENSMUSG00000040987	*Mill2*	MHC I like leukocyte 2	7	3	1.09×10^{-3}
ENSMUSG00000086373	*Gm11978*	Predicted gene 11978	11	3	1.10×10^{-3}
ENSMUSG00000041609	*Ccdc64*	Coiled-coil domain containing 64	5	3	1.11×10^{-3}
ENSMUSG00000037126	*Psd*	Pleckstrin and Sec7 domain containing	19	3	1.16×10^{-3}
ENSMUSG00000035545	*Leng8*	Leukocyte receptor cluster (LRC) member 8	7	3	1.19×10^{-3}
ENSMUSG00000026058	*Khdrbs2*	KH domain containing, RNA binding, signal transduction associated 2	1	3	1.24×10^{-3}
ENSMUSG00000038742	*Angptl6*	Angiopoietin-like 6	9	3	1.34×10^{-3}
ENSMUSG00000089911	*Hiat1*	Hippocampus abundant gene transcript 1	3	3	1.35×10^{-3}
ENSMUSG00000023092	*Fhl1*	Four and a half LIM domains 1	X	3	1.35×10^{-3}
ENSMUSG00000074194	*Zfp791*	Zinc finger protein 791	8	3	1.40×10^{-3}
ENSMUSG00000081616	*Gm14247*	Predicted gene 14247	2	3	1.41×10^{-3}
ENSMUSG00000036430	*Tbcc*	Tubulin-specific chaperone C	17	3	1.42×10^{-3}
ENSMUSG00000003378	*Grik5*	Glutamate receptor, ionotropic, kainate 5 (gamma 2)	7	3	1.52×10^{-3}
ENSMUSG00000032786	*Alas1*	Aminolevulinic acid synthase 1	9	3	1.62×10^{-3}
ENSMUSG00000078816	*Prkcg*	Protein kinase C, gamma	7	3	1.71×10^{-3}
ENSMUSG00000038630	*Zkscan16*	Zinc finger with KRAB and SCAN domains 16	4	3	1.78×10^{-3}
ENSMUSG00000084910	*C630043F03Rik*	RIKEN cdna C630043F03 gene	4	3	1.89×10^{-3}
ENSMUSG00000038555	*Reep2*	Receptor accessory protein 2	18	3	1.92×10^{-3}
ENSMUSG00000089509		Small nucleolar RNA SNORD113/SNORD114 family	12	3	2.00×10^{-3}
ENSMUSG00000052117	*D630039A03Rik*	RIKEN cdna D630039A03 gene	4	3	2.04×10^{-3}
ENSMUSG00000032381	*Fam96a*	Family with sequence similarity 96, member A	9	3	2.10×10^{-3}
ENSMUSG00000058709	*Egln2*	EGL nine homolog 2 (C. Elegans)	7	3	2.15×10^{-3}
ENSMUSG00000071451	*Psmg4*	Proteasome (prosome, macropain) assembly chaperone 4	13	3	2.18×10^{-3}
ENSMUSG00000017724	*Etv4*	Ets variant gene 4 (E1A enhancer binding protein, E1AF)	11	3	2.19×10^{-3}
ENSMUSG00000037243	*Zfp692*	Zinc finger protein 692	11	3	2.20×10^{-3}
ENSMUSG00000087226	*Gm14015*	Predicted gene 14015	2	3	2.27×10^{-3}
ENSMUSG00000068154	*Insm1*	Insulinoma-associated 1	2	3	2.41×10^{-3}
ENSMUSG00000073633	*Fbxo36*	F-box protein 36	1	3	2.44×10^{-3}
ENSMUSG00000020307	*Cdc34*	Cell division cycle 34 homolog (S. Cerevisiae)	10	3	2.67×10^{-3}
ENSMUSG00000075705	*Sepx1*	Selenoprotein X 1	17	3	2.70×10^{-3}
ENSMUSG00000025873	*Faf2*	Fas associated factor family member 2	13	3	2.71×10^{-3}

ENSMUSG00000030500	*Slc17a6*	Solute carrier family 17 (sodium-dependent inorganic phosphate cotransporter), member 6	7	3	2.77×10^{-3}
ENSMUSG00000085838	*Gm13706*	Predicted gene 13706	2	3	2.89×10^{-3}
ENSMUSG00000025478	*Dpysl4*	Dihydropyrimidinase-like 4	7	3	3.01×10^{-3}
ENSMUSG00000090208	*Gm15851*	Predicted gene 15851	1	3	3.17×10^{-3}
ENSMUSG00000047264	*Zfp358*	Zinc finger protein 358	8	3	3.20×10^{-3}
ENSMUSG00000046807	*AI646023*	Expressed sequence AI646023	10	3	3.32×10^{-3}
ENSMUSG00000068798	*Rap1a*	RAS-related protein-1a	3	3	3.36×10^{-3}
ENSMUSG00000064254	*Ethe1*	Ethylmalonic encephalopathy 1	7	3	3.49×10^{-3}
ENSMUSG00000086740	*Gm17029*	Predicted gene 17029	4	3	3.54×10^{-3}
ENSMUSG00000050017	*Pitpnb*	Phosphatidylinositol transfer protein, beta	5	3	3.60×10^{-3}
ENSMUSG00000066829	*Zfp810*	Zinc finger protein 810	9	3	3.64×10^{-3}
ENSMUSG00000018919	*Tm4sf5*	Transmembrane 4 superfamily member 5	11	3	3.81×10^{-3}
ENSMUSG00000053161	*Wdr69*	WD repeat domain 69	1	3	4.00×10^{-3}
ENSMUSG00000089005		U6 spliceosomal RNA	9	3	4.28×10^{-3}
ENSMUSG00000001054	*Rmnd5b*	Required for meiotic nuclear division 5 homolog B (S. Cerevisiae)	11	3	4.41×10^{-3}
ENSMUSG00000019362	*D8Ertd738e*	DNA segment, Chr 8, ERATO Doi 738, expressed	8	3	4.48×10^{-3}
ENSMUSG00000021900	*Btd*	Biotinidase	14	3	4.50×10^{-3}
ENSMUSG00000048039	*Isg20l2*	Interferon stimulated exonuclease gene 20-like 2	3	3	4.72×10^{-3}
ENSMUSG00000053730	*Tmem39b*	Transmembrane protein 39b	4	3	4.99×10^{-3}
ENSMUSG00000039795	*Zfand1*	Zinc finger, AN1-type domain 1	3	4	9.52×10^{-5}
ENSMUSG00000039278	*Pcsk1n*	Proprotein convertase subtilisin/kexin type 1 inhibitor	X	4	1.80×10^{-4}
ENSMUSG00000018634	*Crhr1*	Corticotropin releasing hormone receptor 1	11	4	2.38×10^{-4}
ENSMUSG00000081041	*Nlk-ps1*	Nemo like kinase, pseudogene 1	2	4	3.59×10^{-4}
ENSMUSG00000022376	*Adcy8*	Adenylate cyclase 8	15	4	4.33×10^{-4}
ENSMUSG00000051323	*Pcdh19*	Protocadherin 19	X	4	5.12×10^{-4}
ENSMUSG00000030307	*Slc6a11*	Solute carrier family 6 (neurotransmitter transporter, GABA), member 11	6	4	5.15×10^{-4}
ENSMUSG00000042903	*Foxo4*	Forkhead box O4	X	4	5.47×10^{-4}
ENSMUSG00000006930	*Hap1*	Huntingtin-associated protein 1	11	4	6.06×10^{-4}
ENSMUSG00000008035	*Mid1ip1*	Mid1 interacting protein 1 (gastrulation specific G12-like (zebrafish))	X	4	7.01×10^{-4}
ENSMUSG00000032402	*Smad3*	MAD homolog 3 (Drosophila)	9	4	7.16×10^{-4}
ENSMUSG00000032174	*Icam5*	Intercellular adhesion molecule 5, telencephalin	9	4	7.70×10^{-4}
ENSMUSG00000029623	*Pdap1*	PDGFA associated protein 1	5	4	8.61×10^{-4}
ENSMUSG00000079657	*Rab26*	RAB26, member RAS oncogene family	17	4	9.11×10^{-4}
ENSMUSG00000017754	*Pltp*	Phospholipid transfer protein	2	4	9.49×10^{-4}
ENSMUSG00000031834	*Pik3r2*	Phosphatidylinositol 3-kinase, regulatory subunit, polypeptide 2 (p85 beta)	8	4	9.57×10^{-4}
ENSMUSG00000047613	*A430005L14Rik*	RIKEN cdna A430005L14 gene	4	4	1.05×10^{-3}
ENSMUSG00000087489	*Gm15656*	Predicted gene 15656	8	4	1.05×10^{-3}
ENSMUSG00000051209	*Gpr119*	G-protein coupled receptor 119	X	4	1.06×10^{-3}
ENSMUSG00000029152	*Ociad1*	OCIA domain containing 1	5	4	1.10×10^{-3}
ENSMUSG00000078958	*Atp6ap1l*	Atpase, H+ transporting, lysosomal accessory protein 1-like	13	4	1.11×10^{-3}
ENSMUSG00000036699	*Zcchc12*	Zinc finger, CCHC domain containing 12	X	4	1.13×10^{-3}
ENSMUSG00000026687	*Aldh9a1*	Aldehyde dehydrogenase 9, subfamily A1	1	4	1.15×10^{-3}

ENSMUSG00000032175	*Tyk2*	Tyrosine kinase 2	9	4	1.20×10^{-3}
ENSMUSG00000067370	*B3galt4*	UDP-Gal:betaglcnac beta 1,3-galactosyltransferase, polypeptide 4	17	4	1.20×10^{-3}
ENSMUSG00000023036	*Pcdhgb6*	Protocadherin gamma subfamily A, 11	18	4	1.22×10^{-3}
ENSMUSG00000035285	*Nat14*	N-acetyltransferase 14	7	4	1.25×10^{-3}
ENSMUSG00000017188	*Ccdc56*	Coiled-coil domain containing 56	11	4	1.27×10^{-3}
ENSMUSG00000051497	*Kcnj16*	Potassium inwardly-rectifying channel, subfamily J, member 16	11	4	1.37×10^{-3}
ENSMUSG00000005982	*Nat15*	N-acetyltransferase 15 (GCN5-related, putative)	16	4	1.42×10^{-3}
ENSMUSG00000033335	*Dnm2*	Dynamin 2	9	4	1.42×10^{-3}
ENSMUSG00000062542	*Syt9*	Synaptotagmin IX	7	4	1.49×10^{-3}
ENSMUSG00000071324	*Armc2*	Armadillo repeat containing 2	10	4	1.57×10^{-3}
ENSMUSG00000023020	*2310016M24Rik*	RIKEN cdna 2310016M24 gene	15	4	1.61×10^{-3}
ENSMUSG00000090673	*Gm340*	Predicted gene 340	19	4	1.70×10^{-3}
ENSMUSG00000043811	*Rtn4r*	Reticulon 4 receptor	16	4	1.75×10^{-3}
ENSMUSG00000028976	*Slc2a5*	Solute carrier family 2 (facilitated glucose transporter), member 5	4	4	1.78×10^{-3}
ENSMUSG00000069565	*Dazap1*	DAZ associated protein 1	10	4	1.89×10^{-3}
ENSMUSG00000068299	*1700019G17Rik*	RIKEN cdna 1700019G17 gene	6	4	1.96×10^{-3}
ENSMUSG00000039783	*Kmo*	Kynurenine 3-monooxygenase (kynurenine 3-hydroxylase)	1	4	1.97×10^{-3}
ENSMUSG00000051344	*Plekhm3*	Pleckstrin homology domain containing, family M, member 3	1	4	1.99×10^{-3}
ENSMUSG00000029556	*Hnf1a*	HNF1 homeobox A	5	4	2.05×10^{-3}
ENSMUSG00000024944	*Arl2*	ADP-ribosylation factor-like 2	19	4	2.11×10^{-3}
ENSMUSG00000023909	*Paqr4*	Progestin and adipoq receptor family member IV	17	4	2.15×10^{-3}
ENSMUSG00000001151	*Pcnt*	Pericentrin (kendrin)	10	4	2.17×10^{-3}
ENSMUSG00000027669	*Gnb4*	Guanine nucleotide binding protein (G protein), beta 4	3	4	2.17×10^{-3}
ENSMUSG00000030495	*Slc7a10*	Solute carrier family 7 (cationic amino acid transporter, y+ system), member 10	7	4	2.19×10^{-3}
ENSMUSG00000011096	*Akt1s1*	AKT1 substrate 1 (proline-rich)	7	4	2.20×10^{-3}
ENSMUSG00000032478	*Nme6*	NME/NM23 nucleoside diphosphate kinase 6	9	4	2.29×10^{-3}
ENSMUSG00000028878	*Fam76a*	Family with sequence similarity 76, member A	4	4	2.31×10^{-3}
ENSMUSG00000029291	*Rufy3*	RUN and FYVE domain containing 3	5	4	2.35×10^{-3}
ENSMUSG00000042043	*Tbca*	Tubulin cofactor A	13	4	2.46×10^{-3}
ENSMUSG00000034748	*Sirt6*	Sirtuin 6 (silent mating type information regulation 2, homolog) 6 (S. Cerevisiae)	10	4	2.49×10^{-3}
ENSMUSG00000028443	*Nudt2*	Nudix (nucleoside diphosphate linked moiety X)-type motif 2	4	4	2.56×10^{-3}
ENSMUSG00000037086	*1110059M19Rik*	RIKEN cdna 1110059M19 gene	X	4	2.61×10^{-3}
ENSMUSG00000003948	*Mmd*	Monocyte to macrophage differentiation-associated	11	4	2.67×10^{-3}
ENSMUSG00000038366	*Lasp1*	LIM and SH3 protein 1	11	4	2.72×10^{-3}
ENSMUSG00000069171	*Nr2f1*	Nuclear receptor subfamily 2, group F, member 1	13	4	2.74×10^{-3}
ENSMUSG00000029176	*Anapc4*	Anaphase promoting complex subunit 4	5	4	2.87×10^{-3}
ENSMUSG00000028468	*Rgp1*	RGP1 retrograde golgi transport homolog (S. Cerevisiae)	4	4	2.87×10^{-3}

ENSMUSG00000031134	*Rbmx*	RNA binding motif protein, X chromosome	X	4	2.91×10^{-3}
ENSMUSG00000024621	*Csf1r*	Colony stimulating factor 1 receptor	18	4	2.95×10^{-3}
ENSMUSG00000042524	*Sun2*	Sad1 and UNC84 domain containing 2	15	4	2.96×10^{-3}
ENSMUSG00000004892	*Bcan*	Brevican	3	4	2.97×10^{-3}
ENSMUSG00000070509	*Rgma*	RGM domain family, member A	7	4	3.10×10^{-3}
ENSMUSG00000016637	*Ift27*	Intraflagellar transport 27 homolog (Chlamydomonas)	15	4	3.22×10^{-3}
ENSMUSG00000056211	*R3hdm1*	R3H domain 1 (binds single-stranded nucleic acids)	1	4	3.33×10^{-3}
ENSMUSG00000043857	*Mgat5b*	Mannoside acetylglucosaminyltransferase 5, isoenzyme B	11	4	3.43×10^{-3}
ENSMUSG00000054855	*Rnd1*	Rho family gtpase 1	15	4	3.49×10^{-3}
ENSMUSG00000022433	*Csnk1e*	Casein kinase 1, epsilon	15	4	3.50×10^{-3}
ENSMUSG00000027603	*Ggt7*	Gamma-glutamyltransferase 7	2	4	3.67×10^{-3}
ENSMUSG00000019338	*Zfp687*	Zinc finger protein 687	3	4	3.75×10^{-3}
ENSMUSG00000050627	*Gpd1l*	Glycerol-3-phosphate dehydrogenase 1-like	9	4	3.81×10^{-3}
ENSMUSG00000039754	*Alkbh4*	Alkb, alkylation repair homolog 4 (E. Coli)	5	4	3.84×10^{-3}
ENSMUSG00000022248	*Rad1*	RAD1 homolog (S. Pombe)	15	4	3.91×10^{-3}
ENSMUSG00000037070	*Rbmxl1*	RNA binding motif protein, X linked-like-1	8	4	3.93×10^{-3}
ENSMUSG00000006998	*Psmd2*	Proteasome (prosome, macropain) 26S subunit, non-atpase, 2	16	4	3.97×10^{-3}
ENSMUSG00000027834	*Serpini1*	Serine (or cysteine) peptidase inhibitor, clade I, member 1	3	4	4.21×10^{-3}
ENSMUSG00000033323	*Ctdp1*	CTD (carboxy-terminal domain, RNA polymerase II, polypeptide A) phosphatase, subunit 1	18	4	4.33×10^{-3}
ENSMUSG00000022982	*Sod1*	Superoxide dismutase 1, soluble	16	4	4.37×10^{-3}
ENSMUSG00000084020	*Gm12282*	Predicted gene 12282	11	4	4.39×10^{-3}
ENSMUSG00000029703	*Lrwd1*	Leucine-rich repeats and WD repeat domain containing 1	5	4	4.41×10^{-3}
ENSMUSG00000011658	*Fuz*	Fuzzy homolog (Drosophila)	7	4	4.45×10^{-3}
ENSMUSG00000025735	*Rhbdl1*	Rhomboid, veinlet-like 1 (Drosophila)	17	4	4.50×10^{-3}
ENSMUSG00000029465	*Arpc3*	Actin related protein 2/3 complex, subunit 3	5	4	4.53×10^{-3}
ENSMUSG00000062526	*Mppe1*	Metallophosphoesterase 1	18	4	4.69×10^{-3}
ENSMUSG00000029617	*Ccz1*	CCZ1 vacuolar protein trafficking and biogenesis associated homolog (S. Cerevisiae)	5	4	4.69×10^{-3}
ENSMUSG00000086859	*2810008D09Rik*	RIKEN cdna 2810008D09 gene	11	4	4.74×10^{-3}
ENSMUSG00000038520	*Tbc1d17*	TBC1 domain family, member 17	7	4	4.76×10^{-3}
ENSMUSG00000031558	*Slit2*	Slit homolog 2 (Drosophila)	5	4	4.79×10^{-3}
ENSMUSG00000021792	*5730469M10Rik*	RIKEN cdna 5730469M10 gene	14	4	4.82×10^{-3}
ENSMUSG00000021676	*Iqgap2*	IQ motif containing gtpase activating protein 2	13	4	4.92×10^{-3}
ENSMUSG00000000197	*Nalcn*	Sodium leak channel, non-selective	14	4	4.96×10^{-3}
ENSMUSG00000000247	*Lhx2*	LIM homeobox protein 2	2	4	4.97×10^{-3}

[a]Chr: chromosome. [b]The cluster number corresponds to Fig. 2.
